# Oroya Fever and Verruga Peruana: Bartonelloses Unique to South America

**DOI:** 10.1371/journal.pntd.0002919

**Published:** 2014-07-17

**Authors:** Michael F. Minnick, Burt E. Anderson, Amorce Lima, James M. Battisti, Phillip G. Lawyer, Richard J. Birtles

**Affiliations:** 1 Division of Biological Sciences, University of Montana, Missoula, Montana, United States of America; 2 Department of Molecular Medicine, Morsani College of Medicine, University of South Florida, Tampa, Florida, United States of America; 3 Laboratory of Parasitic Diseases, National Institute of Allergy and Infectious Diseases, National Institutes of Health, Bethesda, Maryland, United States of America; 4 School of Environment and Life Sciences, University of Salford, Salford, United Kingdom; University of California San Diego School of Medicine, United States of America

## Abstract

*Bartonella bacilliformis* is the bacterial agent of Carrión's disease and is presumed to be transmitted between humans by phlebotomine sand flies. Carrión's disease is endemic to high-altitude valleys of the South American Andes, and the first reported outbreak (1871) resulted in over 4,000 casualties. Since then, numerous outbreaks have been documented in endemic regions, and over the last two decades, outbreaks have occurred at atypical elevations, strongly suggesting that the area of endemicity is expanding. Approximately 1.7 million South Americans are estimated to be at risk in an area covering roughly 145,000 km^2^ of Ecuador, Colombia, and Peru. Although disease manifestations vary, two disparate syndromes can occur independently or sequentially. The first, Oroya fever, occurs approximately 60 days following the bite of an infected sand fly, in which infection of nearly all erythrocytes results in an acute hemolytic anemia with attendant symptoms of fever, jaundice, and myalgia. This phase of Carrión's disease often includes secondary infections and is fatal in up to 88% of patients without antimicrobial intervention. The second syndrome, referred to as verruga peruana, describes the endothelialcell-derived, blood-filled tumors that develop on the surface of the skin. Verrugae are rarely fatal, but can bleed and scar the patient. Moreover, these persistently infected humans provide a reservoir for infecting sand flies and thus maintaining *B. bacilliformis* in nature. Here, we discuss the current state of knowledge regarding this life-threatening, neglected bacterial pathogen and review its host-cell parasitism, molecular pathogenesis, phylogeny, sand fly vectors, diagnostics, and prospects for control.

## Methods

Authors were included based upon their relevant expertise in the field. Available databases were searched with key words for applicable articles in both English and Spanish. All cited work was previously peer-reviewed and published elsewhere.

## Janus: The Two Faces of Carrión's Disease

### A brief history

Bartonellosis, or Carrión's disease, is a remarkable bi-phasic illness that manifests as either angiogenic lesions that occur primarily on the skin or as a severe hemolytic anemia. It is caused by *Bartonella bacilliformis*, a bacterium presumed to be transmitted between humans through bites of infected female phlebotomine sand flies, most notably *Lutzomyia verrucarum* [1]–[4]. Humans are the only known reservoir. Carrión's disease is endemic to arid, high-altitude (approximately 600–3200 m) valleys in the Andes Mountains of Peru, Ecuador, and Colombia [5]–[10], coincident with the habitat of the sand fly vector [5]. Evidence for bartonellosis in the region predates Columbus. Skin lesions akin to those typical of bartonellosis are depicted on 2,000-year-old pre-Inca ceramics, and *Bartonella*-like bacteria have been observed in skin lesions taken from the mummified remains of a human sacrifice victim who died over 1,000 years ago [11]. Descriptions of these lesions, referred to as verruga peruana (VP), appear in the journals of Spanish conquistadors who arrived in South America during the 16th century, and in 1630, Gago de Vadillo published the first medical treatise on the disease [12].

Two events inexorably linked with the early history of bartonellosis occurred within 15 years of one another at the end of the 19th century. The first was a dramatic outbreak of hemolytic anemia among workers based in the upper Rimac valley, above Lima, during the construction of a railway line to the mining town of La Oroya in 1871. During the course of a few weeks at least 4,000 men died of what became known as Oroya Fever (OF), although the agent of the syndrome remained elusive. Second, the common etiology of OF and VP was revealed when, in 1885, a medical student named Daniel Carrión injected himself with exudate from a verruga lesion, then developed OF and died [12]. Carrión sacrificed his life to establish the common etiology of VP and OF and is one of the most heroic figures in Peruvian medical history. In honor of his contribution, South American bartonellosis is commonly known as Carrión's disease. Finally, in 1905, Alberto Barton solved the riddle of the etiological agent of Carrión's disease when he observed intracellular bacteria in blood smears from Oroya fever patients. These bacteria were subsequently named *Bartonella bacilliformis* in his honor.

Carrión's disease is most common in Peru, where nearly all cases have occurred over the last 70 years in the Departments of Ancash, Cajamarca, Cuzco, Lima, and Amazonas [13]. Sporadic cases may have also occurred in Chile, Bolivia, and Guatemala [14]. During the last two decades, several outbreaks have occurred at abnormal altitudes and in historically nonendemic regions of Ecuador, Colombia, and Peru (e.g., near sea level in Huaral and Ica, Peru [13], [15]; the Utcubamba River Valley in Amazonas [16]; Urubamba Valley and Sacred Valley of the Incas near Cuzco [17], [18]; and the high Amazonas jungle [19]). In addition, multiple outbreaks have occurred concurrently in endemic regions (e.g., Ancash Region, Peru; Zamora-Chinchipe Province, Ecuador) [5]–[8], [20], [21]. Children have been hardest hit by these outbreaks, with fatality rates of approximately 10% [13], [15], [22]. A 10-fold increase in cases (948 to 10,390) was reported in Peru from 1997–2005 [23], with 52 cases per 100,000 Peruvians in 1999 [13]. Reported rates are considerably higher in endemic regions of Peru, at 242 cases per 100,000 inhabitants (http://www.dge.gob.pe/). An estimate of the at-risk population for Carrión's disease is 1.7 million people in an area of 145,000 km^2^ [15]. In one prospective cohort study of a Peruvian community in an endemic area, an incidence rate of 12.7 per 100 person-years was estimated, reflecting a clustering of 70% of cases within 18% of homes [24].

Factors contributing to the emergence and geographical expansion of *B. bacilliformis* are unclear but include climatic events that favor sand fly propagation, such as increased temperature, humidity, and rainfall during El Niño cycles [21], [25], [26], and possibly additional sand fly species serving as competent vectors (see “Sand fly vectors,” below) [5], [27]–[29]. In short, Carrión's disease is a reemerging public health threat in several South American nations and to travelers who visit these countries [10], [30].

### Oroya fever (OF)

**Figure 1:**
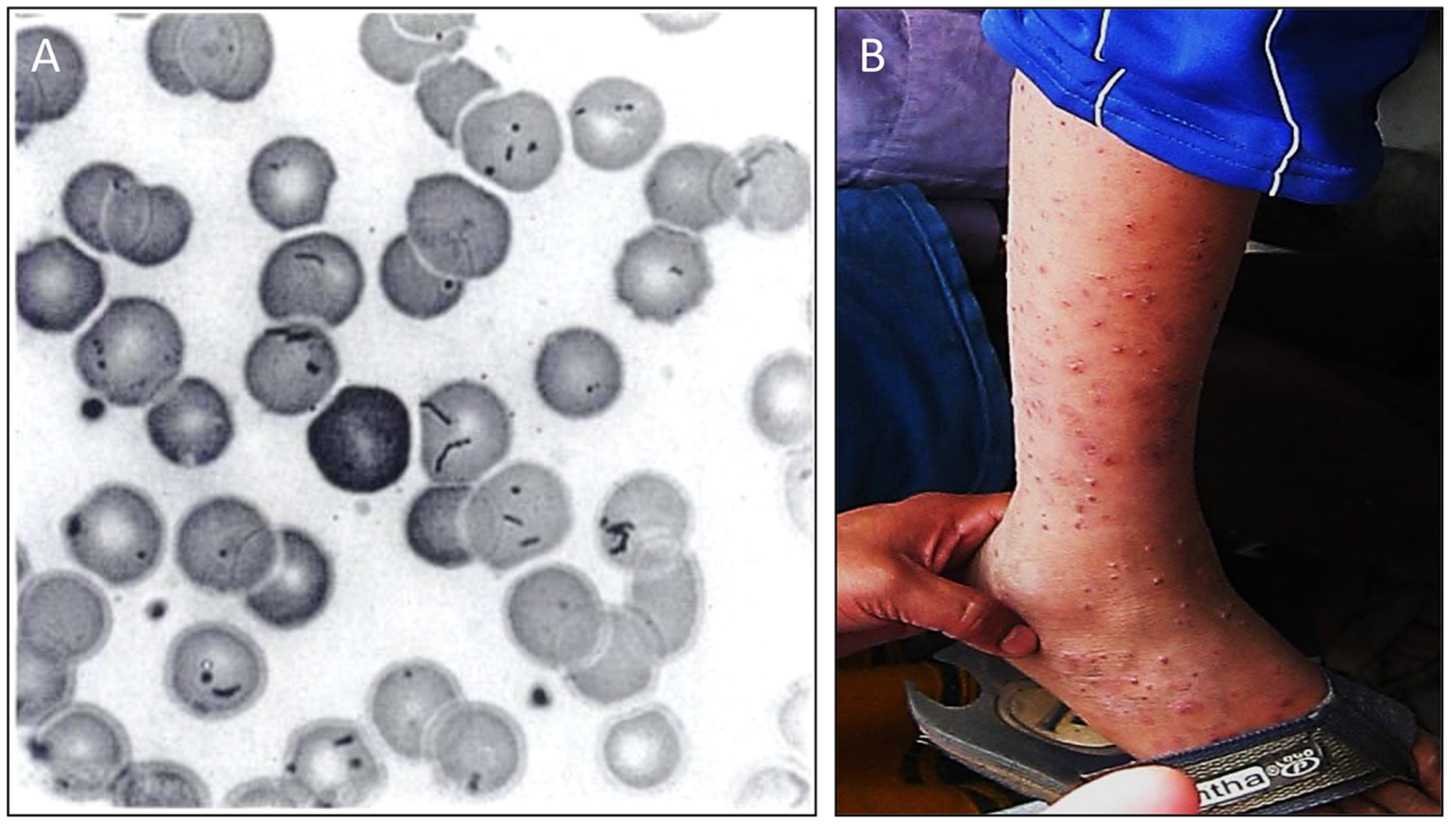
Clinical manifestations of Carrión's disease. (A) Erythrocyte infection during OF, as observed in a blood smear stained with Wright's stain (reprinted by permission from [203]). (B) VP lesions on a child in Peru (reproduced from Future Microbiology 4(6): 743–758 (2009) with permission of Future Medicine, Ltd).

Carrión's disease manifests as two distinct syndromes that occur independently or sequentially. The first phase, OF (also known as the acute, primary or hematic phase), is more common in children (>60% of cases [13], [22], [31]) in both endemic and nonendemic areas, and it is characterized by an acute bacteremia at about 60 days (range of 10–210 days; [32]) following the bite of an infected sand fly. From the inoculation site, *B. bacilliformis* colonizes the entire circulatory system, and nearly every erythrocyte is infected (Figure 1A). OF involves a severe reduction in hematocrit (>80% destruction) and acute hemolytic anemia from splenic culling of infected erythrocytes [33]–[35]. Patients with OF present for 1–4 weeks with an array of symptoms, including pallor, fever, anorexia, malaise, cardiac murmur, myalgia, headache, jaundice, tachycardia, and hepatomegaly. Complications are common during OF, and include high mortality in pregnant women and their unborn children, cardiovascular and neurological problems, respiratory infections, and arthralgia [22], [36]. This phase of disease can be fatal in 40%–88% of infected patients without antimicrobial intervention [2], [5], [12], [14], [32], [36], [37] and is by far the highest reported case-fatality rate for any *Bartonella* species. Secondary infections are also common and potentially life-threatening, and they may arise due to an immune-compromised state that accompanies the disease. These include salmonellosis, toxoplasmosis, malaria, shigellosis, histoplasmosis, and pneumocystosis [5], [14], [37]. Even with appropriate antimicrobial therapy, fatality rates of 9%–11% are typical [13], [15], [38]. A milder form of OF has also been reported with a significantly lower case-fatality rate (0.7%), suggesting that virulence potential and associated disease severity may be strain-dependent [16]. *Bartonella rochalimae*, a distantly related *Bartonella* species isolated from a tourist who visited South America has been reported to cause a relatively mild febrile illness that could be confused with OF. Although associated symptoms are similar, no intraerythrocytic bacteria are observed in blood smears and there is only a slight drop in hematocrit and mild anemia [39].

### Verruga peruana (VP)

The second syndrome (also known as the tissue, chronic, or eruptive phase) involves blood-filled nodular hemangiomas of the skin, termed VP or Peruvian warts (Figure 1B). Tissue involvement results from bacterial invasion of capillary endothelium and generates bacteria-filled vacuoles, termed Rocha Lima inclusions, and localized cellular proliferation, leading to formation of VP [40] (see “Angiogenesis,” below). Verrugae are cutaneous and usually occur on the head and extremities, where they can persist for several weeks to months. Lesions are classified as miliary (multiple red papules of <3 mm in diameter), mular (blood-filled nodules), and diffuse (groups of subdermal nodules >5 mm in diameter) [32]. Although OF occurs at a lower frequency, the eruptive phase is a more common manifestation in inhabitants of endemic regions [16]. The higher incidence of verruga in endemic areas likely reflects the immune status of the population (see “Host immune response, protection,” below). In cases of sequential illness, patients erupt with verrugae roughly 4–8 weeks after resolving OF [41]. VP is rarely fatal, but lesions can bleed or scar the patient, and eruptions can be accompanied by fever and malaise, lymphadenopathy, acute bone and joint pains, headache, and a chronic *Bartonella* infection [2], [22], [32]. The recent discovery of a new *Bartonella* taxon, referred to as *Candidatus* Bartonella ancashi, as the etiological agent of persistent bacteremia and VP in a three-year-old boy living near Caraz, Peru, indicates that species other than *B. bacilliformis* may be responsible for some chronic bartonellosis cases in endemic areas [42]. Whether *Candidatus* Bartonella ancashi can cause OF is unknown.

### Sand fly vectors

*B. bacilliformis* is believed to be transmitted by bites of phlebotomine sand flies to humans living in the high mountain valleys of Peru and neighboring countries of Andean South America. Townsend was the first to advance this view, and he identified *Phlebotomus verrucarum* (now *L. verrucarum*) as the vector of “Verruga” [43]–[45]. Experiments by Townsend and other workers provided strong circumstantial evidence of sand fly vectorship but no conclusive proof.

Having the suspicion based on circumstantial evidence that a suspected vector insect transmits a specific pathogen to man or other animals does not constitute incrimination. One must determine the role the insect plays in the natural history of the pathogen. Killick-Kendrick and Ward [46] outlined five criteria that must be fulfilled to declare with reasonable certainty that a sand fly is a vector of human disease. Applied to the transmission of *B. bacilliformis*, these are:

1. The suspected vector sand fly must bite humans and be present in a place where humans become infected with *B. bacilliformis*.
2. The distribution of the suspected vector should encompass the distribution of the disease in humans, and the sand fly must be sufficiently abundant to maintain pathogen transmission.
3. *B. baciliformis* should be isolated from wild-caught sand flies and be shown to be indistinguishable from the pathogen causing disease in humans in the same area.
4. It should be demonstrated that naturally or experimentally infected flies can maintain the infection through the complete extrinsic life cycle of the pathogen.
5. Experimental transmission of *B. bacilliforms* by bite or some other means would generally be considered conclusive proof that a sand fly is a vector of a given pathogen.

Of these criteria, the first two are fairly easy to satisfy through observations of sand fly behavior in the field, and numerous workers have done so. The remaining three criteria are more difficult to satisfy and must be accomplished through painstaking analysis and experimentation to conclusively incriminate a sand fly species as a vector of *B. bacilliformis*.

Beginning in 1926, Noguchi and associates conducted several experiments in which triturated, wild-caught sand flies from the “Verruga zone” in Peru were inoculated into monkeys to produce infection [47], [48]. Around the same time, Battistini performed a series of experiments in which sand flies collected from the Verruga zone were released into a screened cage with a rhesus monkey. Although this was the first sand fly experiment in which the disease was transmitted by means other than inoculation, the sand flies were not observed and it was not determined whether transmission was by bite [49]–[51]. Between 1937 and 1939, Hertig and associates conducted several feeding experiments in which wild-caught sand flies were either released into small cages with a rhesus monkey, or placed in small feeding cages and strapped to the monkey's shaved belly. Five monkeys became infected as determined by blood culture but no cultures were made from the sand flies [1]. Furthermore, uncertainty remained as to whether transmission was by bite or some other means such as by defecation, as “… there existed opportunities for other types of contact than that of the mouthparts between sand fly and monkey, such as the deposit of fecal matter if such there should be” [1]. Later, these scientists isolated what they said was *B. bacilliformis* from the proboscis of each of two “*Phlebotomus verrucarum*” (*L. verrucarum*) collected from an epidemic focus at Huinco in the Santa Eulalia Valley [1]. Because culture methods used at that time were likely not sufficiently refined to distinguish between *B. bacilliformis* and other *Bartonella* species, there is a lingering question as to whether this was the same pathogen causing disease in humans. However, it is fair to say that Hertig's team was the first to incriminate *L. verrucarum* as a vector of *B. bacilliformis*. Attempts to culture *B. bacilliformis* from sand fly guts were unsuccessful; hence, they were unable to demonstrate any developmental cycle in the sand fly that might explain the mechanism of transmission [1].

**Figure 2:**
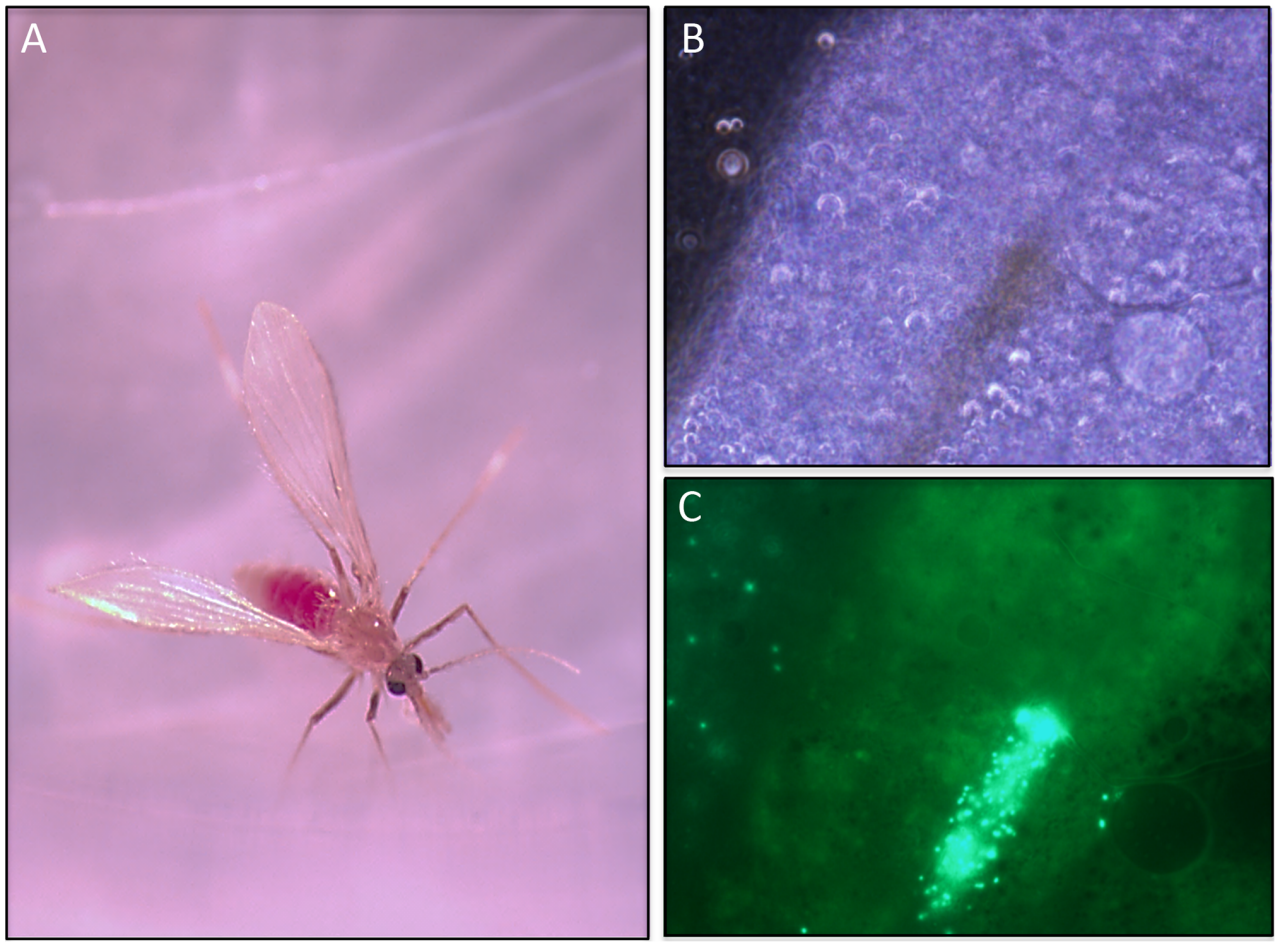
*B. bacilliformis* infection of a phlebotomine sand fly. (A) Female *L. verrucarum* at 16 h post-feeding with an artificial blood feeder containing human blood and GFP-expressing *B. bacilliformis* (low-passage strains 14866 and 14868). (B) Light micrograph of *L. verrucarum* midgut at five days post-feeding on human blood containing GFP^+^ *B. bacilliformis*. Central brown area is residual blood meal. (C) Corresponding UV light micrograph of (B). Note the GFP^+^ *B. bacilliformis* in residual blood meal and elsewhere in the midgut.

In preliminary experiments conducted recently by three of the authors (MFM, JMB, and PGL), *L. verrucarum* from a laboratory colony (Walter Reed Army Institute of Research) were fed through a chick-skin membrane on human blood containing green fluorescent protein (GFP)-labeled *B. bacilliformis*. Serial dissections of putatively infected flies and microscopic examination of their guts under UV over seven days revealed massive proliferation of the bacteria within the blood meal and invasion of red blood cells during the first three days (Figure 2). The infection persisted for at least seven days in the gut of the sand fly. Contrary to what Hertig observed [1], no *B. bacilliformis* were seen on the mouthparts or in any organ other than the midgut. Diuretic droplets deposited on glass cover slips following feeding contained no labeled bacteria, an indication that posterior station transmission via diuresis is unlikely. Transmission by bite during a second blood meal was not attempted, nor were fecal droplets from the digested blood meal examined for *B. bacilliformis* contamination. The mechanism of transmission of *B. bacilliformis* by sand flies is still unclear and is the subject of ongoing investigations.

#### Other vector sand flies

Owing to discrepancies between the distribution of Carrión's disease and *L. verrucarum*, the existence of secondary vectors was suggested [27]. Whereas *L. verrucarum* is limited to altitudes below approximately 3,200 m above sea level *L. peruensis* has been collected in human habitations in endemic villages as high as 3,740 m ASL [27; 2005, Wilfredo Arque, unpublished data, personal communication]. Since Hertig's landmark experiments, substantial evidence has accumulated in Peru to also incriminate *L. peruensis*, which is highly anthropophilic and abundant or dominant in bartonellosis foci in Ancash and Cusc departments, respectively. In studies conducted during an outbreak in the Urubamba Valley, *L. peruensis* in large numbers and a few *L. pescei* were the only sand fly species found. *B. bacilliformis* was identified using PCR in 2% of *L. peruensis* analyzed [17]. In two other foci in Peru, 2%–8% of pools of *L. verrucarum* collected from patient houses in the Caraz area, Ancash Department, and 2% of pools of *L. peruensis* collected from patient houses in villages 70 km south of Cusco, Cusco Department were PCR positive for *B. bacilliformis* (2005, Sofia Romero, unpublished data, personal communication).

Besides *L. verrucarum* and *L. peruensis*, several other sand fly species are suspected vectors of *B. bacilliformis*. Caceres et al., working in the Peruvian provinces of Jaen, San Ignacio and Utcubamba, found *L. robusta* and *L. maranonensis* in great abundance in intradomiciliary habitats and, because of the absence of *L. verrucarum* and *L. peruensis*, considered these as likely vectors of *B. bacilliformis* in associated foci [28]. Elsewhere, on the eastern slope of the Andes in what is known as “Yungas,” or high jungle, Tejada et al. found that *L. serrana*, an avid man-biter, comprised 93.6% of sand flies collected during an investigation of a bartonellosis outbreak in Huamalíes, Huánuco Department [52].

In neighbor Andean countries of Colombia and Ecuador, where *L. verrucarum* and *L. peruensis* are absent, other species are suspected as vectors, but insufficient evidence has been produced to implicate them with certainty. Dominant species in Colombian districts where human bartonellosis has been reported, include *L. gomezi*, *L. panamensis*, and *L. serrana*, all of which are anthropophilic but none of which has ever been associated with *B. bacilliformis* transmission [6]. *L. colombiana* is considered the most likely vector of *B. bacilliformis* in Colombia based on its highly anthropophilic behavior and occurrence in all three departments of the country from which bartonellosis outbreaks have been reported [6]. The species that transmits this pathogen in Ecuador remains unknown [6].

#### Spatial and temporal dynamics of vector sand flies

**Figure 3:**
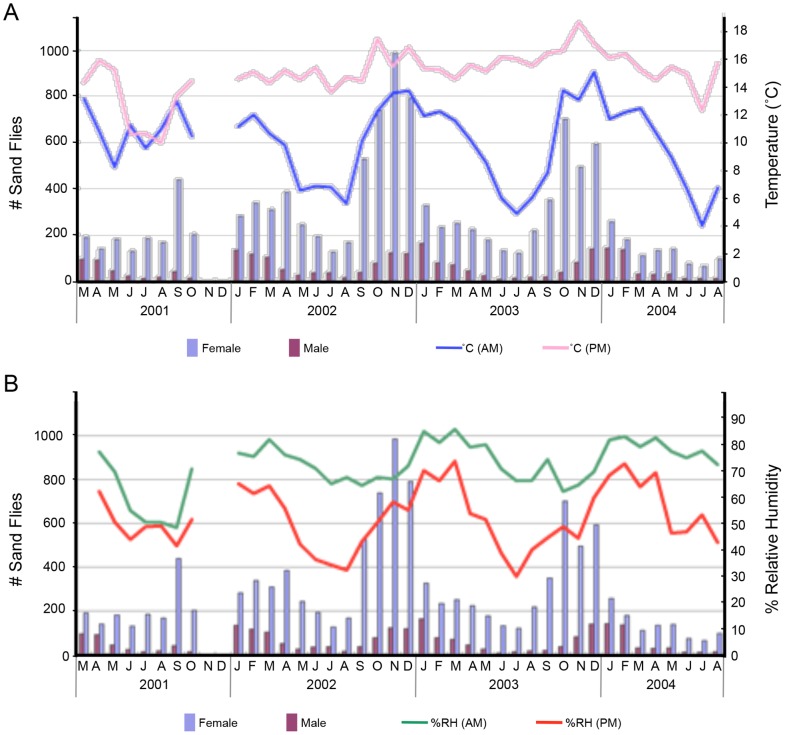
Monthly sand fly collection results from three villages in the Cusco Region, Peru. Results show a unimodal annual population distribution pattern with: (A) corresponding mean morning (blue line) and evening (pink line) temperatures and (B) corresponding mean morning (green line) and evening (red line) relative humidity. Collections were made during two nights per month at case homesteads from March 2001 to August 2004. The data gap between October 2001 and January 2002 is due to a cessation of activity mandated by the Peruvian Ministry of Health.

**Figure 4:**
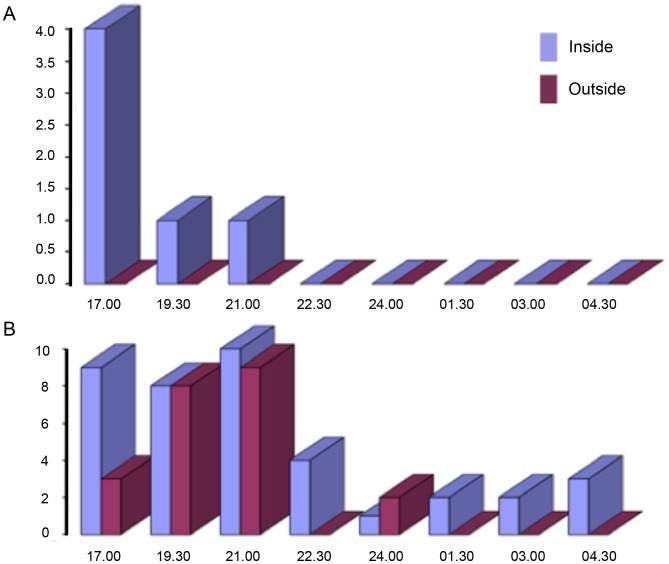
Results of collection-bottle-rotator (CBR) trap collections of sand flies in Peru. Results show that: (A) nightly sand fly activity is limited to early evening (1800–2000 hrs) from March through July, the coldest part of the year, which represents the Peruvian winter, and (B) as nighttime temperatures increase in late August through November (late winter and spring), sand fly activity extends throughout the night. “Inside” and “outside” refer to trap locations within and outside a domicile, respectively.

Three-year longitudinal studies of suspected vector sand flies associated with bartonellosis outbreaks in Ancash and Cusco departments of Peru revealed that both *L. verrucarum* and *L. peruensis* exhibit unimodal annual temporal distribution patterns with lowest population densities occurring in June and July, corresponding to the “winter” dry season; and highest population densities in November, prior to the onset of the “summer” rainy season in December (Figure 3) [53]. It was further determined that sand fly population densities are directly correlated with average minimum ambient temperatures and relative humidity (Figure 3) [53]. As nighttime temperatures and relative humidity increase, sand fly activity also increases (Figure 4) [53]. These findings are consistent with the notion that the height of the *B. bacilliformis* transmission season, March to May, [24], [26] corresponds to the descending side of the seasonal sand fly population peak, February to June, when the aggregate population is oldest and when the highest percentage of flies have had an opportunity to take at least one blood meal and are thus more likely to be infected.

In the Ancash and Cusco regions of Peru, approximately 80% of the flies were captured inside case houses, mostly in sleeping quarters, and 35%–40% of females were blood-fed. Being weak fliers, sand flies breed in close proximity to their blood-meal source, feeding at dusk and during the evening, when ambient temperatures drop and relative humidity rises [53]. *L. verrucarum* and *L. peruensis* both rest inside human dwellings and feed readily indoors, preferentially on humans [24], [53]. Results of bloodmeal analyses conducted in an endemic focus of cutaneous leishmaniasis in Chaute, Lima, showed that *L. peruensis* is more anthropophilic than is *L. verrucarum* [54]. It was also shown that multiple-host blood meals are common, including bloodmeals containing blood from animals only present within the houses mixed with blood from animals only present in nearby shelters, indicating movement of flies from animal shelters to the houses (or vice versa) to finish feeding. People living in endemic villages report that the sand fly bites occur indoors during the evening, directly before and during the hours of sleep, further confirming that transmission occurs inside the home during the evening and the night [24].

As entomological field studies have shown, vectors of infectious diseases, including vectors of bartonellosis, are sensitive to temperature, humidity, wind, and rainfall patterns; therefore, their abundance is influenced by climate variability [24], [26]. During the El Niño weather event that occurred in Peru during late 1997 through mid-1998 weather cycles, the rainy season was extended, and the humidity and minimum monthly temperatures rose. This climatic anomaly allowed potential vector populations to flourish, and unusually high numbers of sand flies were collected [24], [26]. The net result was that major epidemics of bartonellosis erupted in Peru, especially in Caraz and Cusco areas. The impact of weather extremes on vector sand fly ecology and transmission of *B. bacilliformis* warrants thorough study [26].

## Host Cell Parasitism, Angiogenesis, and Immune Response

### Colonization of the host: How, when, and where

**Figure 5:**
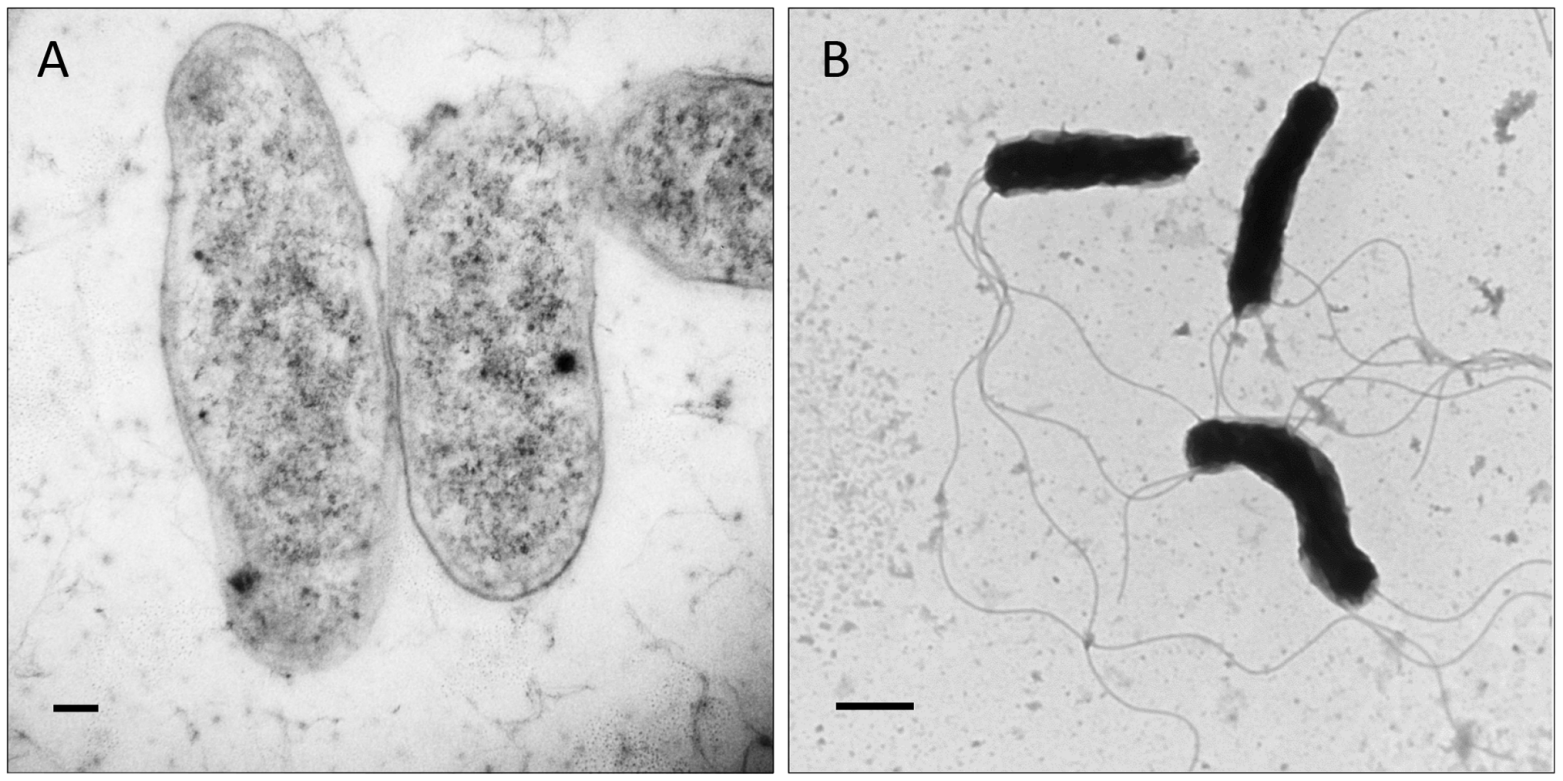
Transmission electron micrographs showing morphology of *B. bacilliformis*. Bacteria were grown three days on heart infusion agar containing 4% sheep erythrocytes and 2% sheep serum at 30°C and 100% relative humidity. Cells were subsequently fixed in 2% glutaraldehyde in cacodylate (pH 7.2), epoxy embedded by standard methods, then sectioned and stained with uranyl acetate (UA) and lead citrate stains. Micrographs show *B. bacilliformis* (strain KC583): (A) from a thin section; (B) applied directly to a grid stained with UA to show flagella. Scale bars represent 100 nm in (A) and 500 nm in (B).

**Figure 6:**
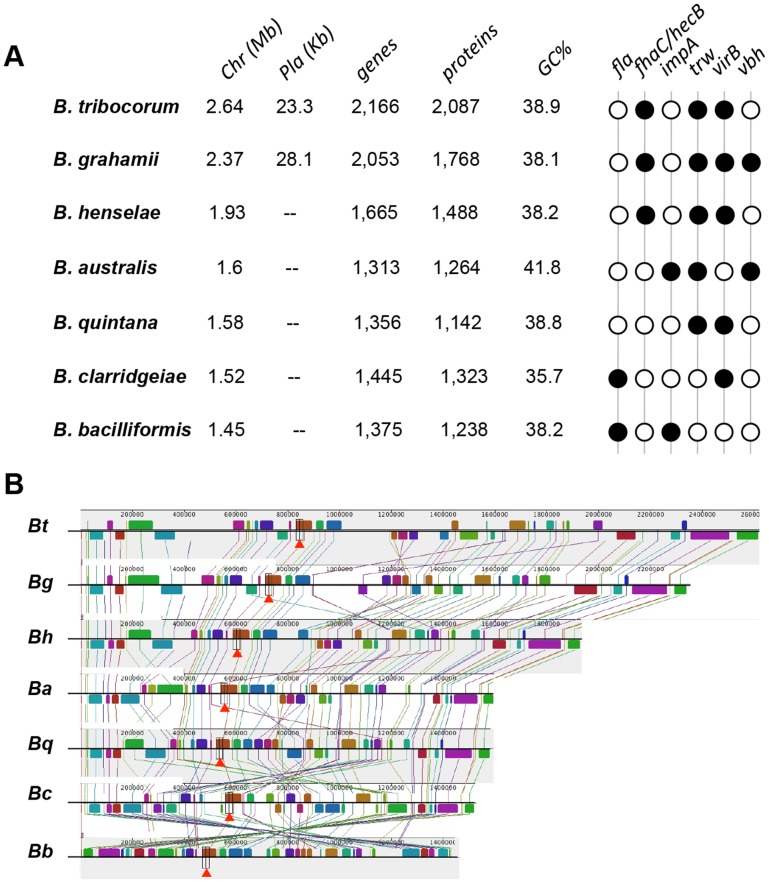
Genomic structure of seven *Bartonella* chromosomes. (A) Chromosomes (Chr) are arranged in size from largest (*B. tribocorum*) to smallest (*B. bacilliformis*). Plasmid (Pla) sizes are listed, if present. *B. quintana*'s genome encodes the least number of proteins (1,142), and *B. clarridgeiae* has the lowest GC% (35.7%). Several virulence-related ORFs have been used to infer phylogeny (*fla – vbh*) and black circles indicate their presence in a particular species. (B) Multiple alignment of seven complete genomes using pM. Location, orientation and position of locally collinear syntenic blocks (LCBs) shared amongst all chromosomes are color-coded and connected by lines. User can analyze location, orientation, and size of LCBs in multiple chromosomes simultaneously (red arrowheads). Local rearrangements, duplications, and inversions are easily identified. Abbreviations correspond to the *Bartonella* species shown in (A).

*Bartonella* species infect an array of mammals with species specificity for their natural reservoir host(s); humans generally serve as incidental hosts. Notable exceptions include *B. bacilliformis* (human reservoir) and *B. quintana* (found in humans and nonhuman primates [55]). Infection of reservoir mammals is generally subclinical with persistent intraerythrocytic bacteremia. *Bartonella* are typically inoculated directly into blood by the bite of an arthropod vector such as fleas, ticks, and sand flies or indirectly when feces of the vector are scratched into the skin. *B. henselae* can also be transmitted via the scratch of a contaminated cat claw. The persistence of bartonellae in the natural host facilitates uptake and transmission by hematophagous arthropod vectors. Colonization of the host's lymphatics and circulatory system may be enhanced by blood flow and perhaps motility in flagellated species. For example, *B. bacilliformis* are highly motile by lophotrichous flagella (Figures 5 and 6), appendages consisting of approximately 42 kDa flagellin subunits that are resistant to protease treatment [56].

### Adherence to host cells

*Bartonella* infections involve interactions with both nucleated cells and erythrocytes. While erythrocyte adherence by different bartonellae is known to be host-specific, adhesion to nucleated cells is considered to be relatively generic. *B. bacilliformis* invades a variety of human cell types, including endothelial cells, dermal fibroblasts, and laryngeal epithelium [57]. Similarly, *B. henselae* and *B. quintana* have been shown to adhere and invade diverse human cell types, including epithelial cells [58], endothelial cells [59], [60], and macrophages [61]. In each case, trimeric autotransporter adhesin proteins (TAAs) are thought to play a leading role in adherence to nucleated host cells [62], [63].

*B. bacilliformis* possesses three *brp* genes encoding *Bartonella* repeat proteins (Brps) [64]. These proteins, along with *Bartonella* adhesin A (BadA) of *B. henselae* and variably expressed outer membrane proteins (Vomps) of *B. quintana*, belong to the TAA family of type V secretion systems [62], [63]. BadA is the best-understood *Bartonella* adhesin and consists of surface filaments with head, neck, and repetitive stalk domains assembled on a C-terminal anchor [65]. BadA's head is crucial for host cell interactions and triggering a proangiogenic response [66]. Although *Bartonella* TAAs share remarkable features and functional similarities, they vary considerably in size, and multiple gene variants are often found in the genome [67]. There are at least two variants of the *badA* gene *in B. henselae*, and four genes encode the Vomps of *B. quintana* (*vompA*–*vompD*) [63]. *B. bacilliformis* Brps share common domains and structural features with TAAs (reviewed in [64]).

The role of BadA has been well studied in *B. henselae*, where it is essential for autoagglutination, adhesion to host cells, and extracellular matrix proteins such as fibronectin and collagens, inhibition of phagocytosis, and induction of a proangiogenic response in host cells [62], [68], [69]. The Vomps of *B. quintana* have also been shown to play a similar role in host cell interaction and autoagglutination [63], [70], [71]. Thus, while the biological role of *B. bacilliformis* Brps has not yet been investigated, it is plausible that the Brps play similar roles in pathogenesis.

Erythrocytes are another major target cell during bartonelloses. Optimal adherence to erythrocytes by *B. bacilliformis* occurs in vitro at approximately six hours after co-incubation [34] and is thought to be energy-dependent [72]. Studies have shown that *B. bacilliformis* and *B. henselae* recognize five or six proteins, respectively, of human erythrocyte membranes [73]. A polar tuft of fibrous projections resembling peritrichous flagella on *B. bacilliformis* was observed to make contact with the erythrocyte membrane during adhesion [72], and antiserum to flagella was shown to significantly reduce *B. bacilliformis* association with red cells as compared to controls [56]. These data suggest that flagella may possess adhesive qualities and/or increase bacteria–host cell collisions. It has been proposed that Trw T4SS and flagella expression are mutually exclusive phenotypes (Figure 6), and the Trw system replaces the erythrocyte adhesion role of flagella among non-flagellated *Bartonella* [74]. Specifically, TrwL and TrwJ adhesins have been shown to result in adherence to erythrocytes. TrwJ1 and TrwJ2 were shown to localize to the bacterial cell surface and serve as species-specific adhesins for erythrocytes [75], [76].

### Invasion of host cells

*Bartonella* species are facultative intracellular bacteria, and growth within host cells is typical. Virulence studies with *B. bacilliformis* and cultured epithelial or endothelial cell monolayers demonstrated that the bacterium can induce host cells to reconfigure the cytoskeleton and enhance uptake. Internalization is significantly reduced (approximately 30% of untreated controls) if actin filament formation is inhibited with cytochalasin D, or if bacteria are pre-treated with anti-*B. bacilliformis* antibodies [57]. These inhibition studies indicate that the bacterium plays an active role during internalization and the process involves a surface-exposed molecule(s) that is accessible to antibody.

Parasitism of erythrocytes (hemotrophy) is an uncommon strategy practiced by a handful of bacterial pathogens, including *Bartonella* and *Anaplasma* species and *Mycoplasma haemofelis* [77]. Hemotrophy is thought to satisfy the absolute requirement of bartonellae for hemin. Erythrocyte invasion by bartonellae is markedly different than entry into other cell types, because red cells are passive, non-endocytotic, and cannot contribute to internalization. Early work showed that three determinants were important for erythrocyte invasion by *B. bacilliformis*, including deformin, flagella, and proteins encoded by an invasion-associated locus (*ialAB*).

**Figure 7:**
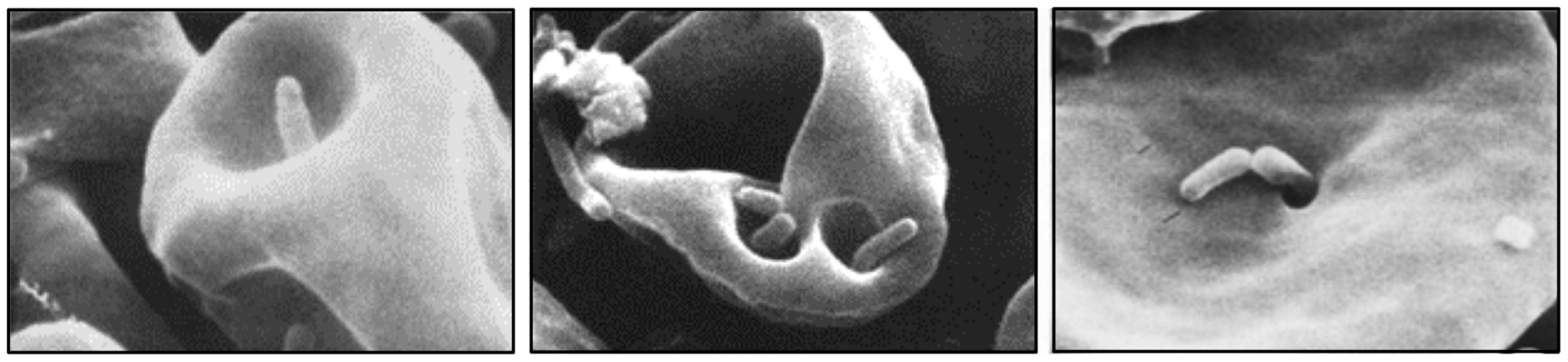
Scanning electron micrographs of deformin-induced invaginations and pits on erythrocyte membranes. *B. bacilliformis* colonization of cell membrane deformations is readily apparent. Reprinted by permission from [34].

Deformin is an extracellular factor that generates trenches and pits in erythrocyte membranes (Figure 7) that resemble those seen on infected red cells [34]. The invaginations are thought to provide entry portals for bacterial colonization. However, even with deformin, bacteria must be motile to access the cytosol [78]. Initial studies showed that deformin was a secreted, homodimeric protein (130 kDa in its native state). Activity was enhanced by pretreatment of erythrocytes with trypsin or neuraminidase and abrogated if cells were pretreated with phospholipase D. Deformin-induced invaginations were reversible by treatment with vanadate or dilauroyl phosphatidylcholine (DLPC), or if intracellular Ca^2+^ levels were increased with ionophores [78], [79]. Subsequent work contradicted earlier results and showed that deformin was a small, hydrophobic molecule of approximately 1400 Da with high affinity for albumin [80]. Finally, a third study implicated a set of 36 kDa proteins in the culture supernatant as being required for maximal activity [81]. Despite these discrepant observations, the nature of deformin and its role in hemotrophy warrant further investigation, especially considering the marked morphological changes the factor produces in erythrocyte membranes.

Flagellum-mediated motility also appears to play an important role in hemotropy. If *B. bacilliformis* is treated with antiflagellin antibodies, in vitro invasiveness for human erythrocytes is nearly abrogated [56]. *B. bacilliformis* also possesses an invasion-associated locus comprised of two genes, *ialA* and *ialB*, that can confer an invasive phenotype on *Escherichia coli* for human red cells in vitro [82]. The *ialA* gene codes for a (di)nucleoside polyphosphate hydrolase that may function to reduce stress-induced dinucleotide “alarmones” encountered in the host cell and therefore enhance pathogen survival [83]. The *ialB* gene codes for a protein with similar molecular mass and considerable amino acid sequence similarity to the adhesion and invasion locus (Ail) protein of *Yersinia enterocolitica* [84] and the resistance to complement killing (Rck) protein of *Salmonella typhimurium* [85]. Both Ail and Rck are implicated in host cell attachment, invasion, and serum resistance. Whether IalA and IalB mediate or facilitate invasion of other host cell types is unknown. A signature-tagged mutagenesis study using *B. birtlesii* identified *ialB* as a gene necessary to establish bacteremia in a mouse model. Moreover, the *ialB* mutant strain displayed a reduced ability to invade erythrocytes in vitro [75]. These results suggest that the *ialAB* locus may be required for optimal intracellular invasion by many *Bartonella* species.

A major difference between *B. bacilliformis* and other common, pathogenic *Bartonella* species, such as *B. henselae* and *B. quintana*, is the absence of VirB T4SS (Figure 6). VirB and the coupling protein VirD4 constitute a machine that delivers T4SS effectors (Beps) to the host cell's cytosol. In turn, translocated Beps subvert cellular functions, including: (1) rearrangement of the actin cytoskeleton, leading to formation of and internalization by invasomes [59], [86]; (2) activation of a pro-inflammatory response via NF-κB [87], [88]; (3) inhibition of apoptosis [88]; and (4) capillary sprout formation in endothelial cells cultured in a collagen matrix [89]. During invasome formation, *B. henselae* are contacted and moved rearward to form an aggregate on the leading lamella of the endothelial cell. The bacterial mass is subsequently internalized by membrane protrusions that are rich in cortical F-actin, ICAM1, and phosphotyrosine. Chemical inhibition studies suggest that invasome activity is actin-dependent and microtubule-independent. Not surprisingly, *B. bacilliformis* cannot trigger invasome formation because it lacks a VirB T4SS.

### Intracellular growth

Bartonellae invade and multiply within cells of their natural hosts. It is thought that seeding of the bloodstream occurs from a primary niche, including the endothelium and migratory cells, such as lymphocytes and phagocytes, or perhaps hematopoietic progenitor cells [74], [90]. Regardless, it appears that these bacteria have increased intracellular growth rates [91]. This is puzzling, considering the absolute requirement of bartonellae for heme and the iron-limiting nature of the intracellular niche. Two independent systems have been described whereby bartonellae bind and transport heme. The hemin-binding proteins (Hbp's) are a group of porin-like, beta-barrel proteins that are surface-exposed [92], [93]. Hbp's are encoded by paralagous gene families [94], and the number of genes varies among species (ranges from three *hbp* genes in *B. bacilliformis* to eight in *B. australis*). *B. bacilliformis* encodes three closely linked, tandem *hbp* genes (e.g., BARBAKC583_1213-1215 and BbINS_05732, 05727, and 05722). Several roles for Hbps have been proposed, including: (1) coating of the bacterium with heme to serve as a nutritive reservoir [93], (2) providing a heme-based antioxidant barrier against reactive oxygen species [93], (3) decreasing oxygen levels around the bacterium to provide a more favorable microaerophilic environment [93], [95], and (4) serving as an adhesin to bind fibronectin, heparin, and HUVECs [96].

*Bartonella* species also employ a more typical hemin utilization (Hut) system consisting of a hemin receptor, an ABC transporter (including a permease, ATPase, and a periplasmic heme-binding protein) and a cytosolic heme-degrading/storage protein [97]. In *B. quintana* and *B. henselae*, the *tonB* gene is located immediately upstream and in opposite orientation to the *hut* locus and encodes TonB, a protein that energizes outer membrane processes, presumably including Hut-mediated heme transport. Interestingly, *B. bacilliformis* lacks an obvious TonB homologue, begging the question as to the nature of its functional surrogate (note: ExbB and ExbD are found in all three bacteria). Regulation of the *hut* locus is accomplished through the iron response regulator (Irr) and promoter regions containing an H-box, a motif known to interact with Irr [97]. Other than Irr's involvement, the hemin transport system in *Bartonella* species is remarkably similar to those of other gram-negative bacteria.

*B. bacilliformis* is generally accepted as nonhemolytic on blood agar. However, incomplete *β*-hemolysis can be observed on thin blood agar plates that are cultured for at least four days. The hemolysin is extracellular and can pass through 0.2 µm filters into underlying medium, where it produces a hemolytic zone that silhouettes the colony's shape [98]. A similar, incomplete *β* hemolytic activity with a delayed appearance (four days' growth) was also observed with *B. elizabethae* [99]. Finally, a contact-dependent hemolysin has been described from *B. bacilliformis* that is proteinaceous, requires prolonged incubation periods, acts independently of deformin, and is most active at 37°C [100]. The molecular nature of the hemolysins and their potential role in virulence have not been investigated thoroughly, to date. It is tempting to speculate that hemolysins participate in escape from vacuoles and cytosolic membranes of host cells.

### Angiogenesis

The clinical manifestation and histological appearance of VP are similar to bacillary angiomatosis (BA), a pathology that can occur in immunocompromised individuals infected with *B. henselae* or *B. quintana* [101]–[103]. VP and BA are characterized by tumor-like lesions or nodules filled with immature capillaries and swollen endothelium due to chronic colonization by bacteria and infiltration of polymorphonuclear leukocytes and macrophages [104], [105]. BA caused by *B. henselae* has been observed primarily on skin, although the bacteria can disseminate and affect other organs such as eyes, liver, and heart [102], [106]. In contrast, VP lesions are rarely observed in organs other than skin and subcutaneous tissue [10]. As in tumor angiogenesis, *Bartonella*-triggered neovascularization follows a series of steps that involve disruption of the normal pattern of the extracellular matrix and basal membrane, endothelial cell migration and proliferation at the site of angiogenic stimuli [107].

In an effort to study angiogenesis induced by *B. bacilliformis*, early studies employed nonhuman primate models, including rhesus monkeys [108]. Resulting angiogenic lesions varied depending on the susceptibility of the monkeys as well as the virulence capacity of the bacteria. Intradermal injection of young rhesus monkeys with *B. bacilliformis* obtained from the blood of a patient with a fatal case of OF gave rise to granulomatous nodules filled with new capillaries similar to VP [47]. Nodules varied in size (0.5–2 cm in diameter and 2–10 mm high), appeared at the site of inoculation within three weeks, and increased in size until 60 days post-inoculation, where they remained stationary before starting to regress [47]. The pathogen was readily found inside angioblasts and endothelial cells of nascent capillaries. Noguchi demonstrated that *B. bacilliformis* that had been passaged in rhesus monkeys, as well as those that were taken from VP tissues led to more severe clinical manifestations of infection [109]. In those test animals, numerous miliary, cherry-red verrugae appeared within one week around the site of inoculation (eyebrows) and were scattered over remote areas such as the groin and legs.

Despite these initial studies, there are few reports describing the molecular basis of *B. bacilliformis*–induced angiogenesis. This is largely due to the lack of an inexpensive in vivo animal model. Although BALB/c mice resist infection by *B. bacilliformis* [110], a SCID/BEIGE murine model for studying *Bartonella* infection may prove useful, but has not been tested in this regard [111]. Garcia et al. investigated the effect of *B. bacilliformis* extracts on human endothelial cells in vitro as well as in an in vivo model of angiogenesis consisting of subcutaneous implantation of sponge discs containing bacterial extracts into adult Sprague-Dawley rats [103]. The study revealed that a mitogenic factor in bacterial extracts caused vascular proliferation in a dose-dependent manner. However, this factor did not induce a pro-inflammatory response as it did not lead to an increase in the number of phagocytes and lymphocytes. A subsequent report found that *B. bacilliformis* GroEL had mitogenic activity toward endothelial cells undergoing serum starvation-induced apoptosis [112], but it did not affect caspase 3 activity. *B. henselae* was also shown to stimulate endothelial cell proliferation, but to a much lesser extent than *B. bacilliformis* extracts [112]. Subsequently, GroEL of *B. henselae* was found in a secreted fraction of the bacterium and caused endothelial cell proliferation and acted by triggering release of intracellular calcium stores [113]. It is possible that GroEL of both species functions as a chaperonin to stabilize a yet unidentified mitogenic factor. However, in contrast to *B. bacilliformis*, an extract from *B henselae* was shown to have an anti-apoptotic factor that reduced apoptosis of endothelial cells by inhibiting caspase 3 and caspase 8 activities [114]. Interestingly, the anti-apoptotic activity was enhanced in the presence of live bacteria.

Our current understanding of *Bartonella*-induced angiogenesis suggests the process involves at least three mechanisms that work synergistically: (1) mitogenic triggering of endothelial cell proliferation, (2) inhibition of apoptosis (contact-dependent or independent), and (3) angiogenic reprogramming of infected host cells (with pro-inflammatory activation of cytokines) [67], [103]. Analysis of VP lesions revealed that infection induces production of VEGF, VEGFR1, VEGFR2, and angiopoietin-2 transcripts [115]. Interestingly, endothelium was shown to produce very little VEGF, whereas overlying epidermis was the major source of VEGF. To our knowledge, the mechanism whereby *B. bacilliformis* induces production of these pro-angiogenic factors has not been investigated, to date. Expression of hypoxia-inducible factor 1 (HIF-1) and subsequent induction of VEGF have been well documented in tumor angiogenesis [116], [117].

*B. henselae* infection studies using in vitro cell culture models and BA tissues showed that bacteria induced HIF-1 activation and VEGF secretion [68], [69]. This angiogenic response was shown to be dependent on expression of BadA and its interaction with β1-integrin mediated by fibronectin binding [62]. Moreover, *B. henselae* infection was shown to trigger a VirB/Bep-dependent angiogenic phenotype in human endothelial cell models. This includes inhibition of endothelial cell apoptosis and a robust induction of capillary sprout formation mediated by BepA [89], [118], and NF-κB-dependent stimulation of a pro-inflammatory response leading to production of IL-8, expression of adhesion molecules such as E-selectin and ICAM-1, and up-regulation of macrophage chemoattractant protein-1 (MCP-1) [105], [119]. It is reasonable to speculate that *B. bacilliformis* does not display effector protein-dependent cell proliferation or anti-apoptotic activity, because it lacks a T4SS. Nevertheless, it may be of interest to determine if *B. bacilliformis* Brps are involved in interaction with β1-integrin and elucidate what role they might play in inducing a pro-angiogenic response.

### Host immune response, protection

Lymphadenopathy is a frequent complication. Immunosuppression is a common manifestation of *B. bacilliformis* infection and probably results from acute anemia and leukocytosis. In fact, a compromised immune state is thought to contribute to the increased incidence of secondary infections seen in infected individuals.

Lifelong humoral immunity apparently results from a *B. bacilliformis* infection [2], [120]. In endemic areas, up to approximately 60% of the population can be seropositive (mainly IgM isotype), and many of these individuals are healthy [121]. Nevertheless, seropositive people who are asymptomatic or post-eruptive for verrugae are often blood culture–positive for *B. bacilliformis* [122]. An extraordinary carrier rate, up to 15%, has been reported in some areas of Peru [123], and it is this infected population that presumably serves as the reservoir [1], [3], [77]. An inverse correlation between patients' chronological age and incidence or disease severity in endemic regions suggests that humoral immunity, as a result of recurring or chronic infections, confers partial immunological protection [24], [36]. Because bartonellae are found in various body fluids and cells, participation by both humoral and cellular arms of the immune system would undoubtedly need to be invoked to resolve the infection. However, for unknown reasons, this does not occur in many afflicted individuals, and the infection is persistent.

## Phylogeny and Genomics: Clues to the Evolution of *B. bacilliformis*

### *Bartonella* comparative genomics

*Bartonella* genomes consist of single, circular chromosomes ranging in size from 1.45 Mb in *B. bacilliformis* to 2.64 Mb in *B. tribocorum*, and are largely collinear with a Guanine+Cytosine (GC) content of 38%–42% [124]. Adenine+Thymidine (AT)-richness of these genomes is a universal attribute of host-associated bacteria. Several *Bartonella* species also harbor circular plasmids [125]–[128], although none have been reported for *B. bacilliformis*. The relatively small size of *Bartonella* genomes is a common attribute among endosymbionts and host-associated bacteria, and possibly reflects genome reduction as a result of decreased selection pressure in the intracellular niche and in the context of a narrower range of hosts [129]. It appears that the number of vertically inherited genes within *Bartonella* genomes is relatively consistent (approximately 1,000), but the number of horizontally acquired (imported) genes varies significantly; thus the genomes of species such as *B. tribocorum* and *B. grahamii*, which both exceed 2 Mb, contain more than 400 imported genes, whereas those of species such as *B. bacilliformis* and *B. quintana*, which are 1.45 and 1.6 Mb respectively, contain 150 or fewer imported genes [127].

Comparative genomics has been used to survey the distribution of coding sequences in extant *Bartonella* species, then correlate this distribution to the phylogenetic juxtapositions of these species as a means to infer patterns of gene acquisition and loss during evolutionary radiation of the genus [124], [130]. The focus of considerable interest is on genes encoding virulence factors that mediate interactions with the host, such as the *virB* and *trw* gene clusters and numerous others described elsewhere in this review, as they are likely key determinants of both host specificity and divergent modes of parasitism that characterize the genus (Figure 6).

### Taxonomy/phylogeny of the genus

The genus *Bartonella* was proposed almost a century ago by Strong to accommodate *B. bacilliformis*, following its description in red blood cells of OF patients by Alberto Barton in 1909 [131]. The advent of 16S rDNA sequencing in the late 1980s suggested that *B. bacilliformis* was genetically similar to the trench fever agent, then called *Rochalimaea quintana* [132], and also a newly emerging pathogen that was subsequently named *Rochalimaea henselae* [133], [134]. The genera *Bartonella* and *Rochalimaea* were later unified with one another [135] and with the genus *Grahamella* [136], taking the name of the longest established taxon, *Bartonella*. Over the past 13 years, the genus has gradually expanded, such that it now includes 31 “validated” species and subspecies (http://www.bacterio.net/index.html) along with many more informal taxa and partially characterized strains. *B. bacilliformis* is the type species of the genus.

Phylogeny of the genus remains somewhat unresolved. The evolutionary relationship of *Bartonella* to *Brucella* and other genera within the *Agrobacterium/Rhizobium* group of α-proteobacteria is well established, but delineation of clusters of species within the genus remains somewhat uncertain. The value of intra-genus phylogenetic inference derived from 16S rDNA alignments was limited due to its conservation amongst *Bartonella* species [137], [138], thus alignments of other loci have been more frequently exploited. The first of these to be widely adopted was *gltA* [139], and phylogenetic inference derived from this locus indicated that *B. bacilliformis* was the most outlying species, in a lineage deeply divergent from other members of the genus. Phylogenies derived from *ftsZ*, *groEL*, and *rpoB* genes also inferred this peripheral position for *B. bacilliformis* [140]. However, the advent of phylogenies derived from alignment of concatenated sequence data from multiple loci cast doubt on *B. bacilliformis*' position as an outlier. Reconstructions by Gundi et al. [141] strongly supported a specific evolutionary relationship with *Bartonella clarridgeiae*, a position that was reaffirmed by Inoue and colleagues in 2010 [142]. This study also inferred that *B. bacilliformis* and *B. clarridgeiae* lay within a well-supported cluster that also contains *B. birtlesii*, *B. bovis*, *B. capreoli*, *B. chomelii*, and *B. schoenbuchensis*.

Whole genome sequence (WGS) data of several *Bartonella* species have now been released, and those of many more are either in draft form or in progress at this writing. Among these, a complete genome is available for one *B. bacilliformis* strain (KC583), whereas data in the form of contigs have been released for a second strain (INS), and projects on nine other strains are currently in progress (http://www.ncbi.nlm.nih.gov/genome/genomes/524). WGS data are being increasingly used to infer a robust phylogeny amongst *Bartonella* species.

Engel and colleagues [130] generated concatenated sequences from 478 loci within genomes of representatives of eight *Bartonella* species (*B. bacilliformis*, *B. clarridgeiae*, *B. grahamii*, *B. henselae*, *B. quintana*, *B. rochalimae*, *B. shoenbuchensis*, *B. tribocorum*, and two unnamed taxa), and from their alignment inferred a phylogeny with four lineages, where *B. bacilliformis* is in a lineage separate from all other *Bartonella*. Most recently, Guy and colleagues [124] described two new, fully resolved genomes (*B. australis* and *B. vinsonii* subspecies *berkhoffii*) and four near-complete genomes (*B. bovis* [2 isolates], *B. vinsonii* subspecies *berkhoffii*, and *B. schoenbuchensis*). The group then compared 428 orthologous core genes amongst the 16 *Bartonella* taxa described above. This phylogeny conflicted with that proposed by Engel et al. [130], in that *B. australis* rather than *B. bacilliformis*, was the earliest diverging species and that *B. bacilliformis* clustered with *B. bovis* and *B. schoenbuchensis*. Subsequent addition of WGS data for an isolate of an informal taxon referred to as “*Bartonella tamiae*” to this dataset and a revision of the genes included did not result in a repositioning of *B. bacilliformis* within the inferred phylogeny, although “*B. tamiae*” replaced *B. australis* as the earliest diverging species [124].

Placement of *B. bacilliformis* within a cluster containing *B. bovis* and *B. schoenbuchensis*, rather than the earliest diverging species of the genus, has led to reevaluation of hypothetical evolutionary processes that have shaped *Bartonella* genomes. The small genome size of *B. bacilliformis* could be explained by reductive genome evolution following a recent host switch; a model akin to that proposed for *B. quintana* [143]. As all other members of the cluster containing *B. bacilliformis* are ruminant-associated, it has been proposed that *B. bacilliformis* descended from an ancestral strain that successfully made a host shift from ruminants to humans [124]. The alpaca (*Vicugna pacos*) and the lama (*Lama glama*), the two most common domesticated ruminant species in the endemic region, are perhaps a source of this ancestral strain. Domestication of these ruminants began some 6,000 years ago, providing a time frame for a hypothetical *B. bacilliformis* host-shift.

### Genetic diversity among *B. bacilliformis* strains

Few studies exploring the genetic diversity of *B. bacilliformis* have been published [144], [145]. These studies employed a variety of different typing methods, including pan-genomic approaches such as amplified fragment-length polymorphism and infrequent restriction site PCR, and/or comparison of nucleotide sequences at loci including *gltA*, *ialB*, and, most frequently, the 16S–23S rDNA internal transcribed spacer (*ITS*). All these approaches have delineated genotypes within the species and have been useful in characterizing the molecular epidemiology of bartonellosis [144], [145]. Most recently, a multilocus sequence typing (MLST) scheme was developed for *B. bacilliformis* [146]. Application of this procedure to 46 isolates collected in Peru yielded interesting findings. Perhaps the most significant was that *B. bacilliformis* may not be a single species; the MLST data provided clear evidence that two isolates recovered from patients with OF had diverged from other *B. bacilliformis* isolates. These isolates are therefore likely members of a novel *Bartonella* genospecies. The two divergent isolates were not epidemiologically linked (i.e., they were obtained nine years apart and from locations that lie 150 km from one another). Furthermore, two additional isolates that are also potential members of this new genospecies have been encountered elsewhere [10]. Taken together, results suggest that novel *B. bacilliformis*-like genospecies have a wide distribution in Peru. The study by Chaloner et al. [146] delineated eight genetic lineages among the isolates surveyed and, in line with many previous studies, these lineages correlated to some degree with the epidemiological provenance of isolates. Thus, strains associated with new foci of Carrión's disease clustered together and were distinct from the most common genotypes, which were encountered across endemic regions of Peru. However, extrapolation of these data to explore the general epidemiology of Carrión's disease may be somewhat premature given the relatively small size and opportunistic nature of the collection examined. This shortfall can only be accurately addressed with systematic surveys, although MLST appears to be an appropriate genotyping method to employ if such studies can be instigated.

In an effort to explore more comprehensively the extent of genetic variation among *B. bacilliformis* strains, we compared the KC583 WGS with genome sequence data available in the form of contigs for the INS isolate (the only genomic data currently available for another *B. bacilliformis* strain [147]), using progressive Mauve (pM). A total of 20 contigs, containing approximately 1,444,079 base pairs of data, were analyzed. Remarkably, our efforts revealed the two datasets differed by only 27 single-nucleotide polymorphisms (SNPs), with no evidence of any indels or repeat motifs. We mapped the position of these 27 SNPs, finding that 19 lay in putative open reading frames. Of these, 13 were nonsynonymous and two introduced a stop codon. Taken together, these observations indicate that the two *B. bacilliformis* strains, despite sharing no specific epidemiological relationship (being isolated roughly 50 years apart from patients inhabiting geographically remote locations) possess, for all intents and purposes, indistinguishable genomes. Such is the extraordinary nature of these findings that their credibility must be questioned; the upcoming release of genomic data from an additional nine *B. bacilliformis* isolates will provide a broader insight into intraspecies genomic diversity and put the results described above into better context.

## Diagnostics and Identification

Diagnostic symptoms of OF include an extraordinarily low erythrocyte count (approximately 500,000/mm^3^) [33], [77], [148] and numerous infected erythrocytes in blood smears prepared with Wright's, Giemsa, or eosin/thiazine stains [32], [121] (Figure 1A). Blood smears have served as a standard diagnostic tool for Carrión's disease for decades, due to low cost and ease of use. However, the test suffers from low sensitivity, despite its high specificity [4]. Diagnoses can also be made based on blood culture, PCR, immunological tests, or a combination thereof (see below). Diagnosis of VP is made by the occurrence of cutaneous angiomatous skin lesions. As in BA, the histopathology of verrugae reveal bacteria, albeit with difficulty [40], when sectioned and stained with Warthin-Starry silver stain [149] or Giemsa stain [32]. A patient's age (especially <5 years old) and whether bartonellosis has recently occurred within the family are also useful predictors of a *B. bacilliformis* infection [24].

### Growth requirements and physiology

Bartonellae can be isolated from patient blood by preparing samples with a lysis-centrifugation method [150] or a freeze-thaw protocol [151]. Primary isolates of *Bartonella* species have also been obtained using a BACTEC blood culture system (Becton Dickinson) and *Bartonella* alphaproteobacteria growth medium (BAPGM) [152], [153]. Homogenized tissues may also be used as an inoculum for blood plates or endothelial cell monolayers [154].

The slow growth rate of *B. bacilliformis* (approximately7-hour generation time in vitro [34]) can complicate identification and requires that isolates be cultured several days to visualize colonies. Most bartonellae are microaerophilic, capnophilic (5% CO_2_), and grown at approximately 37°C, whereas *B. bacilliformis* requires cooler temperatures (25°C–30°C) and ambient CO_2_ for culture. In addition, humidity of the incubator should be near 100%. Like other *Bartonella* species, *B. bacilliformis* cannot synthesize protoporphyrin IX (PPIX) or heme (Fe^2+^-PPIX), and the essential nutrient is obtained from hemin (Fe^3+^-PPIX), hemoglobin, and host erythrocytes. Interestingly, *Bartonella* cannot utilize heme-rich scavenger molecules of the host, such as lactoferrin or transferrin, unlike many pathogens. Commonly used growth media for *Bartonella* species include chocolate agar, hemin agar, or various blood agars (for recipes, see [155]). The basal medium of these formulations varies and includes *Brucella* medium, trypticase soy, GC, heart infusion, or Columbia agars. Media supplements often include erythrocytes (sheep, rabbit, etc.), hemoglobin, IsoVitalex (Becton Dickinson), and serum (sheep). The medium should be pH 7.0–7.5 for optimal growth.

### Colony and cellular morphology, structure

*B. bacilliformis* is a gram-negative, non–acid fast, pleomorphic coccobacillus. Bacteria stain well with Giemsa or Gimenez stains. Most cells measure 0.5 µm wide by 1.0 µm long. Electron micrographs of *B. bacilliformis* are shown in Figure 5. *B. bacilliformis* colonies are typically very small (initially pinpoint but growing to 0.2–1 mm in diameter after several days), round, and lenticular with an even margin and a raised center in older colonies, and range from translucent to opaque. Although colonies from primary isolates can take weeks to become visible, growth is much more rapid (three to seven days to maximal size) in subsequent passages. Colony color can vary on different media but is typically clear to creamy white. *B. bacilliformis* is highly motile by unipolar, lophotrichous flagella, a phenotype shared with *B. clarridgeiae* and *B. rochalimae* [39], [56], [156]. Twitching motility, as observed in low-passage *B. henselae* and *B. quintana* [58], [134], has not been reported. Capsules and endospores are not present in *B. bacilliformis*, in keeping with other *Bartonella* species.

On the whole, the cellular architecture and composition of bartonellae are typical of gram-negative bacteria [157]. Notable exceptions include the unusual cellular fatty acids (CFAs) (see “Cellular Fatty Acid Analysis,” below) and architecture and immunogenicity of its lipopolysaccharide (LPS). A “deep rough” LPS chemotype (little if any O-chain polysaccharide is present), was first reported for *B. bacilliformis* [158], [159]. Later, similar chemotypes were reported for *B. quintana* [160] and *B. henselae* [161]. Further investigation of *B. henselae*'s LPS showed that it contains a unique inner oligosaccharide core of two keto-deoxyoctulosonic acids (KDOs) and one glucose molecule [161]. Its pentaacyl lipid A backbone is also unusual; instead of the typical glucosamine sugars, it contains 2,3-diamino-2,3-dideoxy-glucose dissacharide (GlcN3N) with both sugars phosphorylated. Interestingly, lipid A of *B. henselae*'s LPS possesses a long-chain fatty acid (C_26:0_ or C_28:0_) similar to other intracellular pathogens, including *Legionella* and *Chlamydia* species [161]. It is tempting to speculate that *B. henselae*'s LPS is representative of all *Bartonella*, especially considering the uniform LPS chemotype for the genus. Finally, work has shown that *Bartonella* endotoxin is considerably less toxic relative to LPS from other gram-negative bacteria, and its activation of TLR-4 pathways is significantly (1,000–10,000-fold) lower than *Salmonella*'s LPS [161]. In fact, recent work has shown that *B. quintana* LPS is a potent antagonist of TLR-4-mediated activation of human monocytes by *E. coli* LPS [162]. Whether a relatively low level of endotoxicity and TLR-4 antagonist activity holds for *B. bacilliformis*' LPS is unknown, but would not be surprising.

The cellular and subcellular protein content of *Bartonella* species has been analyzed by several groups over the years. Early work showed that the *B. bacilliformis* outer membrane fraction contains 14 proteins of 11.2 to 75.3 kDa in mass [159], as compared to nine surface-exposed proteins in *B. henselae* [163]. The proteomes of whole cells and subcellular fractions of *B. quintana* and *B. henselae* have been analyzed by two-dimensional gel electrophoresis and MALDI-TOF-MS of the protein spots. For example, two studies identified 44 [164] and 53 [165] proteins associated with the *B. henselae* outer membrane fractions. A third study analyzed the *B. quintana* membrane subproteome and found 60 proteins [166]. Finally, analysis of *B. henselae*'s total proteome identified 191 different proteins (431 spots) [167]. Unfortunately, a comprehensive analysis of the *B. bacilliformis* proteome has not been done, to date.

### Biochemical tests

Bartonellae are nonfermentative, aerobic (microaerophilic) bacteria with a relatively unremarkable physiology. Thus, conventional biochemical tests are not particularly helpful in presumptive identification of species. One problem with using standard biochemical tests on *Bartonella* species is that hemin is normally not included in the medium, and results must be judged cautiously. Notwithstanding this caveat, standard biochemical tests show that *B. bacilliformis* is strictly aerobic and does not utilize carbohydrates by preformed or de novo enzymes. Tests for oxidase, Voges-Proskauer, catalase, indole production, nitrate reduction, urease activity, hippurate hydrolysis, succinate, alkaline phosphatase, tetrathionate reductase, pyrazinamidase, tributyrin, *o*-nitrophenyl-*β*-D-galactoside, esculin hydrolysis, and arginine dihydrolase are all negative [136], [156], [168], [169]. However, tests for peptidase activity on L-proline and L-lysine (acid) are useful in presumptive, differential identification of *B. bacilliformis*, which is negative for proline and positive for lysine utilization [136], [156], [168], [170]. Screens are typically done in multitest formats designed for anaerobes, such as the MicroScan Rapid Anaerobe identification panel (Dade Behring).

### Polymerase chain reaction (PCR)

PCR is sensitive and specific for identifying *Bartonella* species and can be used to confirm a presumptive identification. PCR can be done directly on affected tissues and/or blood or on enrichment cultures [171], [172]. In early work, PCR was used to amplify the 16S rRNA gene or fragments to generate a sequencing template. Resulting data were compared to known 16S rRNA gene sequences [154], [173]–[175]. However, the utility of the 16S RNA gene as a means of differentiating *Bartonella* species was limited due to conservation [137], [138]. More useful PCR-based strategies for genus and species identification have been described for serine protease (*htrA*) [176], *gltA* [139], [177], divisome protein (*ftsZ*) [178], riboflavin synthase (*ribC*) [179], NADH dehydrogenase gamma subunit (*nuoG*) [180], heme-binding protein A/Pap31 (*hbpA/pap31*) [181], tmRNA (*ssrA*) [182], RNA polymerase beta subunit (*rpoB*) [183], and the 16S–23S rRNA *ITS* [138], [184]. One recent report employed PCR, with *rpoB* as the target, and pyrosequencing of the resulting amplicons for rapid species identification, including *B. bacilliformis* [185]. Another used *ialB* as a PCR target and showed high sensitivity and specificity in 15 patients with OF [186]. Finally, a recent study demonstrated the utility of collecting dried-blood spots from patients in endemic areas, followed by quantitative PCR (qPCR). Results showed that 24.6% of 65 children studied were positive by qPCR on spots, whereas only 3% were blood-culture positive [187].

### Cellular Fatty Acid (CFA) analysis

CFA analysis by gas chromatography has been used to identify *Bartonella* to the genus level. This is made possible by the group's atypical fatty acid composition. For example, predominant fatty acids of bartonellae include *cis*-11-octadecanoic acid (C_18:1*ω7c*_) (approximately 54%) and hexadecanoic acid (C_16:0_) (approximately 20%). However, *B. bacilliformis* is unusual in that it contains little octadecanoic acid (C_18:0_) (approximately 2%) compared to *B. elizabethae*, *B. henselae*, *B. clarridgeiae*, and *B. quintana* (approximately 20%). *B. bacilliformis* also has an unusually high amount (approximately 20%) of *cis-1*1-hexadecanoic acid (C_16:1*ω7c*_) relative to other *Bartonella* species (typically <1%) [99], [150], [156], [188], [189].

### Immunological tests

Immunological tests are, for the most part, sensitive and specific for Carrión's disease. However, positive results must be judged cautiously, as they may not indicate an active infection, but rather a persistent humoral immune response. The indirect fluorescence antibody (IFA) test has been used as a specific and sensitive diagnostic tool [190]. In addition, recent work with ELISAs containing recombinant LppB (43-kDa lipoprotein) showed a high degree of sensitivity and specificity in 27 confirmed patients with either chronic or acute disease [191]. Finally, an immunoblot-based protocol incorporating proteins from sonicated *B. bacilliformis* showed good sensitivity with sera from ten cases of acute disease and very high sensitivity using sera from 32 cases of chronic disease [192]. Likewise, work by Maguiña et al. showed a high degree of sensitivity in immunoblots probed with nine and 11 sera from patients with acute and chronic disease, respectively [193], [194]. It is clear that immunological tests are useful diagnostic tools for Carrión's disease, especially when combined with other techniques such as PCR and blood culture. However, immunodiagnostics for this disease are relatively underdeveloped.

## Current Status of Treatment, Prevention and Control

### Syndrome-dependent courses of therapy

Analysis of antimicrobial susceptibilities of four strains of *B. bacilliformis* showed that the bacterium is extremely sensitive to a wide array of drugs in vitro, including aminoglycosides, beta-lactams, macrolides, tetracyclines, fluoroquinolones, chloramphenicol, clorimoxazole, and rifampin [195]. However, outcomes of antimicrobial therapy in patients with Carrión's disease have not been as clear-cut because of several confounding factors, including patient history, immunosuppression, frequent secondary infections, and persistent or recurring bartonellosis despite therapeutic intervention.

Antimicrobial therapy for Carrión's disease is predicated on whether disease is acute or chronic. Acute disease has been treated with chloramphenicol for decades [2], [14], [193], [196]–[199], sometimes in combination with other antimicrobials [193]. One apparently effective mixture is chloramphenicol plus a beta-lactam [197]. The choice of chloramphenicol was undoubtedly made popular due to its effectiveness, low cost, and broad spectrum of activity against common co-infectious agents, such as salmonellae [37], [193]. Tetracyclines (doxycycline), beta-lactams (ampicillin, penicillin G), macrolides (erythromycin, roxithromycin), trimethoprim-sulfamethoxazole, and fluoroquinolones (norfloxacin, ciprofloxacin) have been used as alternatives to treat OF [15], [41], [196], [197]. Although ciprofloxacin holds great promise for treating acute cases, it should be used judiciously, as resistance mutants arise rapidly in vitro [200], [201], and intrinsic resistance to the drug has been reported in strains that were obtained prior to widespread use of fluoroquinolones [202]. Interestingly, in addition to antimicrobial therapy, blood transfusions have been employed for treating severe anemia of OF [36], [203].

Chronic disease is usually treated with rifampin, although streptomycin is also effective and was the drug-of-choice prior to 1975 [5], [193], [197], [199]. Of note is that VP does not respond to either chloramphenicol or penicillin treatment [15], [32]. Ciprofloxacin, azithromycin, and sultamicillin, combined with deflazacort, have been used as alternatives to treat VP [4], [5], [204].

### Prevention and control measures

No vaccine is currently available for *B. bacilliformis*. Early work investigated a composite vaccine prepared from four strains of the bacterium that were grown axenically and formalin-inactivated (0.4% formalin in physiological saline) [205]. Immunizations were administered (one to three times subcutaneously) to 22 men stationed for one month at a Peruvian military outpost located in the “verruga zone”’ near Matucana, Peru. Nineteen of the men seroconverted during the study. Twelve men (55% of test group) became blood-culture positive over the course of exposure and eventually presented with symptoms of bartonellosis that were, for the most part, relatively mild. Unvaccinated control groups, prior to and after the study, showed approximately 75% (anecdotal) and 90% infection rates, respectively, with many incapacitated due to severity of illness [205]. Although preliminary in nature, this pilot study suggests that antibodies generated against formalin-killed *B. bacilliformis* reduce disease severity in humans but do not provide full protection against infection. Reasons for the partial protection are unclear, but may be due to strain-specific antigenic variation, a humoral immune response bias against the vaccine formulation, or modification of important protective antigens by formalin cross-linkage.

In the absence of a vaccine, control of the sand fly vector has been the fallback course of action in endemic areas. As early as World War II, Peruvian army personnel were provided with bed nets and given Vaseline-citronella ointments as a means of warding off sand flies [205]. Early work in endemic areas of Peru showed that DDT insecticide (5% w/v in kerosene) sprayed in and around dwellings was effective at killing sand flies and kept the insects at very low levels for up to four months [206]. The reduction of sand fly numbers “to the vanishing point” using DDT also correlated to a “virtual cessation of bartonellosis” in construction camps within endemic areas of Peru, although exact numbers were not reported [206]. More recent, unpublished results of sand fly control using residual pyrethroid insecticides in homes near Ancash, Peru are promising, as the duration of control was increased to a year (as reported in [4]). Moreover, others have reported good success with residual pyrethroids against old-world sand flies [207]. In any case, control measures using insecticides may prove to be challenging, especially considering that *Lutzomyia* larvae develop in largely unknown terrestrial habitats, and adult flies, when not resting in houses or animal sheds, dwell in deep cracks and crevices during daylight hours; sites that are not readily accessible to superficial application of insecticides.

In Colombia, other interventions including pyrethroid-impregnated bednets and curtains were used as practical means of controlling sand flies entering houses [208]. In Italy and Burkina Faso, permethrin-impregnated curtains reduced the numbers of endophilic phlebotomine sand flies by 90% and 95%, respectively [209], [210]. However, because peak biting activity occurs shortly after sundown and prior to the time when human inhabitants usually retire for the night, impregnated bednets and curtains may have minimal effect in interrupting sand fly–to–human transmission [211]. Furthermore, it has been shown that sand flies can pass through the mesh of an impregnated bednet and bite a host before succumbing to insecticide exposure (Personal communication, Dr. Edgar Rowton, unpublished data). Nevertheless, if the use of impregnated bednets and curtains can shorten the lives of sand flies, and thereby suppress the vector population, the risk of infection may be reduced [211]. Varying results of field studies dictate the need for trials against specific target vectors before adopting this type of intervention as a means of control for *B. bacilliformis* vectors.

Field trials conducted in Israel and Kenya showed that sand flies will feed on sugar sprayed on plants in the flies' natural habitats, suggesting a possible means for controlling sand flies with toxic baits [212]. This finding prompted a pilot study in Kenya to control sand flies resting in rodent burrows and eroded termitaria by spraying vegetation with sugar solution containing *Bacillus sphaericus* and a dye to reveal that the flies had fed on the sugar [213]. Marked flies were caught entering the rodent burrows and termitaria, and the number of flies emerging from the rodent burrows declined significantly [213]. More recently, scientists at Louisiana State University evaluated several chemical insecticide-treated sugar baits and concluded that they could be efficacious in controlling adult sand fly populations with targeted use in specific habitats [214].

In many foci, sand flies are closely associated with rodents and have been recovered entering and exiting rodent burrows (personal observation, PGL). Burrows of many rodents are the suspected habitats of the immature stages [215]. In fact, sand fly larvae have been observed feeding on rodent feces inside a burrow [216]. Both adult and immature stages benefit from the microhabitat created within rodent burrows [211]. Recently, novel research has focused on sand fly control methods that capitalize on the close ecological interaction between sand flies and rodents. Feed-through or systemic insecticides such as ivermectin or spinosad incorporated into rodent baits have been shown to cause 100% mortality of bloodfeeding sand flies for at least one week [217], [218]. In follow-on studies, it was shown that rodents fed on fipronil produced feces that were toxic to coprophagous larvae for up to 21 days. An approach using rodent baits containing fipronil could significantly suppress vector populations that originate in the vicinity of rodent populations, eliminating the portion of the adult population that feeds on the rodents and immature stages that feed on the toxic feces [219].

## Future Prospects

In many respects, Carrión's disease is a “poster-child” for neglected tropical diseases. The illness is endemic to remote, rural areas of South America, and afflicted populations are typically indigent and lack ready access to transportation, modern means of communication, and healthcare. Not surprisingly, few people outside the Andes region are even aware of the disease and the potential public health crisis posed by its spread. Climatic change and vectoring of Carrión's disease by alternate sand fly vectors have undoubtedly contributed to the spread and re-emergence of *B. bacilliformis*. While immediate remediation of climate change is not realistic, improved sand fly control measures in and around homes, especially during the rainy season, and enhanced surveillance and formation of a multinational database to follow the epidemiology of Carrión's disease in endemic and proximal, nonendemic areas of South America is achievable and clearly warranted. Once identified, an adequate response to the outbreak will require more effective-yet-inexpensive diagnostics that can be done rapidly in rural settings with minimal training and modest laboratory equipment. Solid-phase immunodiagnostics (e.g., ELISA, fluorescence microscopy) seem especially well suited for development in the near-term and could be used in tandem with blood smears to rapidly diagnose Carrión's disease. Patient samples (e.g., dried blood spots as in [187]) could then be submitted to regional reference labs for confirmation of presumptive identification using blood culture and/or PCR-based techniques. Fortunately, once *B. bacilliformis* is diagnosed, it is susceptible to several antimicrobials and treatable. Nevertheless, in view of the logistic problems surrounding patient inaccessibility to healthcare, communication, and transportation to urban areas, the development of a protective vaccine is clearly warranted. To this end, both B- and T-cell epitopes of *B. bacilliformis* must be identified to allow for the generation of a protective, multivalent subunit vaccine that invokes both humoral and cellular immune responses. The human-restricted reservoir and infection bode well for the vaccine-mediated eradication of Carrión's disease. The key obstacle to overcome to realize this goal is a considerable quantity of capital for research and development.

**Key Learning Points**

- *B. bacilliformis* is a sand fly–vectored bacterium responsible for an emerging infectious disease of humans that is endemic to South America.
- *B. bacilliformis* is the most virulent *Bartonella* species and causes two disparate syndromes; a life-threatening, acute infection of erythrocytes (OF) and a comparatively benign, chronic infection of the vascular endothelium (VP).

- The genome of *B. bacilliformis* has undergone extensive reduction in response to specialization related to its human-only parasitism and reservoir.
- Virulence determinants are understudied but include adhesins (e.g., *Bartonella* repeat proteins), flagella, a deformation factor for erythrocyte membranes (deformin), an invasion-associated locus encoding two proteins (*ialAB*), a mitogenic protein that may facilitate angiogenesis, and two heme-acquisition systems (Hut and Hbp's).
- Improved diagnostics, better sand fly control measures, enhanced monitoring and surveillance for outbreaks, and a protective vaccine are urgently needed to control Carrión's disease in South America.

**Top Five Papers in Field**

1. **Foundations:** Benson LA, Kar S, McLaughlin G, Ihler GM (1986) Entry of *Bartonella bacilliformis* into erythrocytes. Infect Immun 54: 347–353.
2. **Angiogenesis:** Minnick MF, Smitherman LS, Samuels DS (2003) Mitogenic effect of *Bartonella bacilliformis* on human vascular endothelial cells and involvement of GroEL. Infect Immun 71: 6933–6942.
3. **Epidemiology:** Chamberlin J, Laughlin LW, Romero S, Solórzano N, Gordon S, et al. (2002) Epidemiology of endemic *Bartonella bacilliformis*: a prospective cohort study in a Peruvian mountain valley community. J Infect Dis 186: 983–990.
4. **Phylogeny:** Chaloner GL, Ventosilla P, Birtles RJ (2011) Multi-locus sequence analysis reveals profound genetic diversity among isolates of the human pathogen *Bartonella bacilliformis*. PLoS Negl Trop Dis 5: e1248.
5. **Control:** Sanchez Clemente N, Ugarte-Gil CA, Solórzano N, Maguiña C, Pachas P, et al. (2012) *Bartonella bacilliformis*: a systematic review of the literature to guide the research agenda for elimination. PLoS Negl Trop Dis 6: e1819.

## References

[1] Hertig M (1942) Phlebotomus and Carrión's Disease. Am J Trop Med Hyg S1-22: 2–81.

[2] Weinman D (1965) The bartonella group. In: Dubos RJ, Hirsch JG, editors. Bacterial and Mycotic Infections of Man. Philadelphia: Lippincott. pp. 775–785.

[3] Birtles RJ, Canales J, Ventosilla P, Alvarez E, Guerra H, et al (1999) Survey of *Bartonella* species infecting intradomicillary animals in the Huayllacallán Valley, Ancash, Peru, a region endemic for human bartonellosis. Am J Trop Med Hyg 60: 799–805. 10344656 10.4269/ajtmh.1999.60.799

[4] Sanchez Clemente N, Ugarte-Gil CA, Solórzano N, Maguiña C, Pachas P, et al (2012) *Bartonella bacilliformis*: a systematic review of the literature to guide the research agenda for elimination. PLoS Negl Trop Dis 6: e1819. 23145188 10.1371/journal.pntd.0001819 PMC3493376

[5] Maguiña C, Gotuzzo E (2000) Bartonellosis. New and old. Infect Dis Clin North Am 14: 1–22. 10738670 10.1016/s0891-5520(05)70215-4

[6] Alexander B (1995) A review of bartonellosis in Ecuador and Colombia. Am J Trop Med Hyg 52: 354–359. 7741177 10.4269/ajtmh.1995.52.354

[7] Amano Y, Rumbea J, Knobloch J, Olson J, Kron M (1997) Bartonellosis in Ecuador: serosurvey and current status of cutaneous verrucous disease. Am J Trop Med Hyg 57: 174–179. 9288812 10.4269/ajtmh.1997.57.174

[8] Cooper P, Guderian R, Orellana P, Sandoval C, Olalla H, et al (1997) An outbreak of bartonellosis in Zamora Chinchipe Province in Ecuador. Trans R Soc Trop Med Hyg 91: 544–546. 9463663 10.1016/s0035-9203(97)90019-5

[9] Cooper P, Guderian R, Paredes W, Daniels R, Perera D, et al (1996) Bartonellosis in Zamora Chinchipe province in Ecuador. Trans R Soc Trop Med Hyg 90: 241–243. 8758062 10.1016/s0035-9203(96)90229-1

[10] Lydy SL, Eremeeva ME, Asnis D, Paddock CD, Nicholson WL, et al (2008) Isolation and characterization of *Bartonella bacilliformis* from an expatriate Ecuadorian. J Clin Microbiol 46: 627–637. 18094131 10.1128/JCM.01207-07 PMC2238110

[11] Allison MJ, Pezzia A, Gerszten E, Mendoza D (1974) A case of Carrión's disease associated with human sacrifice from the Huari culture of Southern Peru. Am J Phys Anthropol 41: 295–300. 4606100 10.1002/ajpa.1330410212

[12] Schultz MG (1968) A history of bartonellosis (Carrión's disease). Am J Trop Med Hyg 17: 503–515. 4876803 10.4269/ajtmh.1968.17.503

[13] Pachas P (2000) Epidemiologia de la bartonelosis en el Peru. Módulos Técnicos, Oficina General de Epidemiología-Insituto Nacional de Salud. Lima, Peru. 83 p.

[14] Gray GC, Johnson AA, Thornton SA, Smith WA, Knobloch J, et al (1990) An epidemic of Oroya fever in the Peruvian Andes. Am J Trop Med Hyg 42: 215–221. 2316791 10.4269/ajtmh.1990.42.215

[15] Maguiña Vargas C (1998) Bartonellosis o enfermedad de Carrión. Nuevos aspectos de una vieja enfermedad. Lima, Peru: AFA Editores Importadores. 195 p.

[16] Kosek M, Lavarello R, Gilman RH, Delgado J, Maguiña C, et al (2000) Natural history of infection with *Bartonella bacilliformis* in a nonendemic population. J Infect Dis 182: 865–872. 10950782 10.1086/315797

[17] Ellis BA, Rotz LD, Leake JA, Samalvides F, Bernable J, et al (1999) An outbreak of acute bartonellosis (Oroya fever) in the Urubamba region of Peru, 1998. Am J Trop Med Hyg 61: 344–349. 10463692 10.4269/ajtmh.1999.61.344

[18] Montoya M, Maguiña C, Vigo B, Caparo R, Briceño E, et al (1998) Brote epidémico de enfermedad de Carrión en el Valle Sagrado de los Incas (Cuzco). Bol Soc Peru Med Int 11: 170–176.

[19] Maco V, Maguiña C, Tirado A, Maco V, Vidal JE (2004) Carrión's disease (Bartonellosis bacilliformis) confirmed by histopathology in the high forest of Peru. Rev Inst Med Trop Sao Paulo 46: 171–174. 15286824 10.1590/s0036-46652004000300010

[20] Solano L, Morocho L, Bueno C, Cesar V, Villalta MC, et al (1993) Investigación de Bartonelosis en el Valle de Puchka, Provincia de Huari, Ancash-Perú. Rev Per Med Trop 7: 13–25.

[21] Huarcaya Castilla E, Chinga Alayo E, Chavez P, Juan M, Chauca Carhuajulca J, et al (2004) Influencia del fenómeno de El Niño en la epidemiología de la bartonelosis humana en los departamentos de Ancash y Cusco entre 1996 y 1999. Rev Med Hered 15: 4–10.

[22] Huarcaya E, Maguiña C, Torres R, Rupay J, Fuentes L (2004) Bartonelosis (Carrion's disease) in the pediatric population of Peru: an overview and update. Braz J Infect Dis 8: 331–339. 15798808 10.1590/s1413-86702004000500001

[23] Pachas P (2005) Situación de la enfermedad de Carrión en el Peru. International Conference of Human Bartonellosis March 21–23, 2005; Lima, Peru.

[24] Chamberlin J, Laughlin LW, Romero S, Solórzano N, Gordon S, et al (2002) Epidemiology of endemic *Bartonella bacilliformis*: a prospective cohort study in a Peruvian mountain valley community. J Infect Dis 186: 983–990. 12232839 10.1086/344054

[25] Chinga-Alayo E, Huarcaya E, Nasarre C, del Aguila R, Llanos-Cuentas A (2004) The influence of climate on the epidemiology of bartonellosis in Ancash, Peru. Trans R Soc Trop Med Hyg 98: 116–124. 14964812 10.1016/s0035-9203(03)00017-8

[26] Zhou J, Lau WK-M, Masuoka PM, Andre RG, Chamberlin J, et al (2002) El Niño helps spread bartonellosis epidemics in Peru. Eos Trans Am Geophys Union 83: 157–161.

[27] Caceres AG (1993) Geographic distribution of *Lutzomyia verrucarum* (Townsend, 1913) (Diptera, Psychodidae, Phlebotaminae), vector of human bartonellosis in Peru. Rev Inst Med Trop Sao Paulo 35: 485–490. 7997750 10.1590/s0036-46651993000600002

[28] Caceres AG, Galati EA, Le Pont F, Velasquez C (1997) Possible role of *Lutzomyia maranonensis* and *Lutzomyia robusta* (Diptera: Psychodidae) as vectors of human bartonellosis in three provinces of region nor Oriental del Marañon, Peru. Rev Inst Med Trop Sao Paulo 39: 51–52. 9394538 10.1590/s0036-46651997000100011

[29] Chomel BB, Boulouis HJ, Breitschwerdt EB, Kasten RW, Vayssier-Taussat M, et al (2009) Ecological fitness and strategies of adaptation of *Bartonella* species to their hosts and vectors. Vet Res 40: 29. 19284965 10.1051/vetres/2009011 PMC2695021

[30] Matteeli A, Castelli F, Spinetti A, Bonetti F, Graifenberghi S, et al (1994) Short report: verruga peruana in an Italian traveler from Peru. Am J Trop Med Hyg 50: 143–144. 8116804 10.4269/ajtmh.1994.50.143

[31] Montoya M (2003) Características clínicas, epidemiológicas, laboratoriales y factores de riesgo en pacientes con bartonellosis aguda complicada. [Tesis de maestría]. Lima: Facultad de Medicina, Universidad Peruana Cayetano Heredia.

[32] Maguiña C, Guerra H, Ventosilla P (2009) Bartonellosis. Clin Dermatol 27: 271–280. 19362689 10.1016/j.clindermatol.2008.10.006

[33] Reynafarje C, Ramos J (1961) The hemolytic anemia of human bartonellosis. Blood 17: 562–578. 13741078

[34] Benson LA, Kar S, McLaughlin G, Ihler GM (1986) Entry of *Bartonella bacilliformis* into erythrocytes. Infect Immun 54: 347–353. 3770946 10.1128/iai.54.2.347-353.1986 PMC260167

[35] Garcia-Caceres U, Garcia FU (1991) Bartonellosis. An immunodepressive disease and the life of Daniel Alcides Carrión. Am J Clin Pathol 95: S58–66. 2008885

[36] Breña Chávez JP, Maguiña Vargas CP, Hernandez Diaz HR, Castillo Diaz ME, Pozo Tovar WE (2006) Bartonelosis aguda en niños: estudio de 32 casos en el Instituto Especializado de Salud del Niño y el Hospital Nacional Cayetano Heredia (período 1993–2003). Rev Med Hered 17: 122–131.

[37] Cuadra MS (1956) Salmonellosis complication in human bartonellosis. Tex Rep Biol Med 14: 97–113. 13337733

[38] Maguiña C (1993) Estudio clinico de 145 casos de bartonelosis en el Hospital Nacional Cayetano Heredia [Tesis doctoral]. Lima: Facultad de Medicina, Universidad Peruana Cayetano Heredia.

[39] Eremeeva ME, Gerns HL, Lydy SL, Goo JS, Ryan ET, et al (2007) Bacteremia, fever, and splenomegaly caused by a newly recognized *Bartonella* species. N Engl J Med 356: 2381–2387. 17554119 10.1056/NEJMoa065987

[40] Arias-Stella J, Lieberman PH, Erlandson RA, Arias-Stella JJ (1986) Histology, immunohistochemistry, and ultrastructure of the verruga in Carrión's disease. Am J Surg Pathol 10: 595–610. 2428261 10.1097/00000478-198609000-00002

[41] Bass JW, Vincent JM, Person DA (1997) The expanding spectrum of *Bartonella* infections. I. Bartonellosis and trench fever. Pediatr Infect Dis J 16: 2–10. 9002093 10.1097/00006454-199701000-00003

[42] Blazes DL, Mullins K, Smoak BL, Jiang J, Canal E, et al (2013) Novel *Bartonella* agent as cause of verruga peruana. Emerg Infect Dis 19: 1111–1114. 23764047 10.3201/eid1907.121718 PMC3713980

[43] Townsend CH (1913) A *Phlebotomus* the practically certain carrier of verruga. Science 38: 194–195. 17740112 10.1126/science.38.971.194

[44] Townsend CH (1913) Preliminary characterization of the vector of verruga, *Phlebotomus verrucarum* sp. nov. Insecutor Inscitiae Menstruus 1: 107–108.

[45] Townsend CH (1914) The Relation between lizards and *Phlebotomus verrucarum* as indicating the reservoir of verruga. Science 40: 212–214. 17800307 10.1126/science.40.1023.212

[46] Killick-Kendrick R, Ward RD (1981) Ecology of *Leishmania*. Workshop No. 11. Parasitology 82: 143–152.

[47] Noguchi H, Battistini TS (1926) Etiology of Oroya fever: I. Cultivation of *Bartonella bacilliformis* . J Exp Med 43: 851–864. 19869166 10.1084/jem.43.6.851 PMC2131137

[48] Noguchi H, Shannon RC, Tilden EB, Tyler JR (1929) Etiology of Oroya fever: XIV. The insect vectors of Carrión's disease. J Exp Med 49: 993–1008. 19869598 10.1084/jem.49.6.993 PMC2131598

[49] Battistini TS (1927) Estudio sobre la verruga. Bol Direc Salubr Pub., 2^nd^ Sem. 1926; Lima. pp. 191–197.

[50] Battistini TS (1929) Estudios sobre la verruga peruana. La Acción Médica, Lima, Jan.

[51] Battistini TS (1931) La verrue peruvienne: sa transmission par le Phlébotome. Rev Sud-Amer Med Chir 2: 719–724.

[52] Tejada A, Vizcarra H, Perez J, Caceres A, Quispe J, et al (2003) Estudio clinico epidemiológico de bartonelosis humana en el valle del Monzón, Huamalíes, Huánuco. An Fac Med 64: 211–217.

[53] Lawyer PG, Andre RG, Fernandez R, Carrasco J, Curo JL, et al (2005) Spatial and temporal dynamics of *Lutzomyia peruensis* (Diptera:Psychodidae) in an epidemic focus of human bartonellosis, Proceedings of the Fifth International Symposium on Phlebotomine Sand Flies. Archives De l'Institut Pasteur De Tunis 82 (Special Issue) 53.

[54] Ogusuku E, Perez JE, Paz L, Nieto E, Monje J, et al (1994) Identification of bloodmeal sources of *Lutzomyia* spp. in Peru. Ann Trop Med Parasitol 88: 329–335. 7944678 10.1080/00034983.1994.11812873

[55] Li H, Liu W, Zhang GZ, Sun ZZ, Bai JY, et al (2013) Transmission and maintenance cycle of *Bartonella quintana* among rhesus macaques, China. Emerg Infect Dis 19: 297–300. 23347418 10.3201/eid1902.120816 PMC3563275

[56] Scherer DC, DeBuron-Connors I, Minnick MF (1993) Characterization of *Bartonella bacilliformis* flagella and effect of antiflagellin antibodies on invasion of human erythrocytes. Infect Immun 61: 4962–4971. 8225570 10.1128/iai.61.12.4962-4971.1993 PMC281270

[57] Hill EM, Raji A, Valenzuela MS, Garcia F, Hoover R (1992) Adhesion to and invasion of cultured human cells by *Bartonella bacilliformis* . Infect Immun 60: 4051–4058. 1398917 10.1128/iai.60.10.4051-4058.1992 PMC257435

[58] Batterman HJ, Peek JA, Loutit JS, Falkow S, Tompkins LS (1995) *Bartonella henselae* and *Bartonella quintana* adherence to and entry into cultured human epithelial cells. Infect Immun 63: 4553–4556. 7591104 10.1128/iai.63.11.4553-4556.1995 PMC173653

[59] Dehio C, Meyer M, Berger J, Schwarz H, Lanz C (1997) Interaction of *Bartonella henselae* with endothelial cells results in bacterial aggregation on the cell surface and the subsequent engulfment and internalisation of the bacterial aggregate by a unique structure, the invasome. J Cell Sci 110: 2141–2154. 9378764 10.1242/jcs.110.18.2141

[60] Brouqui P, Raoult D (1996) *Bartonella quintana* invades and multiplies within endothelial cells in vitro and in vivo and forms intracellular blebs. Res Microbiol 147: 719–731. 9296106 10.1016/s0923-2508(97)85119-4

[61] Musso T, Badolato R, Ravarino D, Stornello S, Panzanelli P, et al (2001) Interaction of *Bartonella henselae* with the murine macrophage cell line J774: infection and proinflammatory response. Infect Immun 69: 5974–5980. 11553533 10.1128/IAI.69.10.5974-5980.2001 PMC98724

[62] Riess T, Andersson SG, Lupas A, Schaller M, Schäfer A, et al (2004) *Bartonella* adhesin a mediates a proangiogenic host cell response. J Exp Med 200: 1267–1278. 15534369 10.1084/jem.20040500 PMC2211922

[63] Zhang P, Chomel BB, Schau MK, Goo JS, Droz S, et al (2004) A family of variably expressed outer-membrane proteins (Vomp) mediates adhesion and autoaggregation in *Bartonella quintana* . Proc Natl Acad Sci U S A 101: 13630–13635. 15347808 10.1073/pnas.0405284101 PMC518805

[64] Kaiser PO, Riess T, O'Rourke F, Linke D, Kempf VA (2011) *Bartonella* spp.: throwing light on uncommon human infections. Int J Med Microbiol 301: 7–15. 20833105 10.1016/j.ijmm.2010.06.004

[65] Linke D, Riess T, Autenrieth IB, Lupas A, Kempf VA (2006) Trimeric autotransporter adhesins: variable structure, common function. Trends Microbiol 14: 264–270. 16678419 10.1016/j.tim.2006.04.005

[66] Kaiser PO, Riess T, Wagner CL, Linke D, Lupas AN, et al (2008) The head of *Bartonella* adhesin A is crucial for host cell interaction of Bartonella henselae. Cell Microbiol 10: 2223–2234. 18627378 10.1111/j.1462-5822.2008.01201.x

[67] O'Rourke F, Schmidgen T, Kaiser PO, Linke D, Kempf VA (2011) Adhesins of *Bartonella* spp. Adv Exp Med Biol 715: 51–70. 21557057 10.1007/978-94-007-0940-9_4

[68] Kempf VA, Volkmann B, Schaller M, Sander CA, Alitalo K, et al (2001) Evidence of a leading role for VEGF in *Bartonella henselae*-induced endothelial cell proliferations. Cell Microbiol 3: 623–632. 11553014 10.1046/j.1462-5822.2001.00144.x

[69] Kempf VA, Lebiedziejewski M, Alitalo K, Wälzlein JH, Ehehalt U, et al (2005) Activation of hypoxia-inducible factor-1 in bacillary angiomatosis: evidence for a role of hypoxia-inducible factor-1 in bacterial infections. Circulation 111: 1054–1062. 15723970 10.1161/01.CIR.0000155608.07691.B7

[70] Schulte B, Linke D, Klumpp S, Schaller M, Riess T, et al (2006) *Bartonella quintana* variably expressed outer membrane proteins mediate vascular endothelial growth factor secretion but not host cell adherence. Infect Immun 74: 5003–5013. 16926391 10.1128/IAI.00663-06 PMC1594870

[71] Müller NF, Kaiser PO, Linke D, Schwarz H, Riess T, et al (2011) Trimeric autotransporter adhesin-dependent adherence of *Bartonella henselae*, *Bartonella quintana*, and *Yersinia enterocolitica* to matrix components and endothelial cells under static and dynamic flow conditions. Infect Immun 79: 2544–2553. 21536788 10.1128/IAI.01309-10 PMC3191982

[72] Walker TS, Winkler HH (1981) *Bartonella bacilliformis*: colonial types and erythrocyte adherence. Infect Immun 31: 480–486. 7012005 10.1128/iai.31.1.480-486.1981 PMC351807

[73] Iwaki-Egawa S, Ihler GM (1997) Comparison of the abilities of proteins from *Bartonella bacilliformis* and *Bartonella henselae* to deform red cell membranes and to bind to red cell ghost proteins. FEMS Microbiol Lett 157: 207–217. 9418257 10.1111/j.1574-6968.1997.tb12775.x

[74] Dehio C (2008) Infection-associated type IV secretion systems of *Bartonella* and their diverse roles in host cell interaction. Cell Microbiol 10: 1591–1598. 18489724 10.1111/j.1462-5822.2008.01171.x PMC2610397

[75] Vayssier-Taussat M, Le Rhun D, Deng HK, Biville F, Cescau S, et al (2010) The Trw type IV secretion system of *Bartonella* mediates host-specific adhesion to erythrocytes. PLoS Pathog 6: e1000946. 20548954 10.1371/journal.ppat.1000946 PMC2883598

[76] Deng HK, Le Rhun D, Le Naour E, Bonnet S, Vayssier-Taussat M (2012) Identification of *Bartonella* Trw host-specific receptor on erythrocytes. PLoS ONE 7: e41447. 22848496 10.1371/journal.pone.0041447 PMC3406051

[77] Kreier JP, Ristic M (1981) The biology of hemotrophic bacteria. Annu Rev Microbiol 35: 325–338. 7027903 10.1146/annurev.mi.35.100181.001545

[78] Mernaugh G, Ihler GM (1992) Deformation factor: an extracellular protein synthesized by *Bartonella bacilliformis* that deforms erythrocyte membranes. Infect Immun 60: 937–943. 1541567 10.1128/iai.60.3.937-943.1992 PMC257577

[79] Xu YH, Lu ZY, Ihler GM (1995) Purification of deformin, an extracellular protein synthesized by *Bartonella bacilliformis* which causes deformation of erythrocyte membranes. Biochim Biophys Acta 1234: 173–183. 7696292 10.1016/0005-2736(94)00271-p

[80] Derrick SC, Ihler GM (2001) Deformin, a substance found in *Bartonella bacilliformis* culture supernatants, is a small, hydrophobic molecule with an affinity for albumin. Blood Cells Mol Dis 27: 1013–1019. 11831868 10.1006/bcmd.2001.0475

[81] Hendrix LR, Kiss K (2003) Studies on the identification of deforming factor from *Bartonella bacilliformis* . Ann NY Acad Sci 990: 596–604. 12860696 10.1111/j.1749-6632.2003.tb07433.x

[82] Mitchell SJ, Minnick MF (1995) Characterization of a two-gene locus from *Bartonella bacilliformis* associated with the ability to invade human erythrocytes. Infect Immun 63: 1552–1562. 7890422 10.1128/iai.63.4.1552-1562.1995 PMC173188

[83] Cartwright JL, Britton P, Minnick MF, McLennan AG (1999) The IalA invasion gene of *Bartonella bacilliformis* encodes a (de)nucleoside polyphosphate hydrolase of the MutT motif family and has homologs in other invasive bacteria. Biochem Biophys Res Commun 256: 474–479. 10080922 10.1006/bbrc.1999.0354

[84] Miller VL, Bliska JB, Falkow S (1990) Nucleotide sequence of the *Yersinia enterocolitica ail* gene and characterization of the Ail protein product. J Bacteriol 172: 1062–1069. 1688838 10.1128/jb.172.2.1062-1069.1990 PMC208537

[85] Heffernan EJ, Wu L, Louie J, Okamoto S, Fierer J, et al (1994) Specificity of the complement resistance and cell association phenotypes encoded by the outer membrane protein genes *rck* from *Salmonella typhimurium* and *ail* from *Yersinia enterocolitica* . Infect Immun 62: 5183–5186. 7927803 10.1128/iai.62.11.5183-5186.1994 PMC303245

[86] Rhomberg TA, Truttmann MC, Guye P, Ellner Y, Dehio C (2009) A translocated protein of *Bartonella henselae* interferes with endocytic uptake of individual bacteria and triggers uptake of large bacterial aggregates via the invasome. Cell Microbiol 11: 927–945. 19302579 10.1111/j.1462-5822.2009.01302.x

[87] Fuhrmann O, Arvand M, Göhler A, Schmid M, Krüll M, et al (2001) *Bartonella henselae* induces NF-kappaB-dependent upregulation of adhesion molecules in cultured human endothelial cells: possible role of outer membrane proteins as pathogenic factors. Infect Immun 69: 5088–5097. 11447190 10.1128/IAI.69.8.5088-5097.2001 PMC98604

[88] Schmid MC, Schulein R, Dehio M, Denecker G, Carena I, et al (2004) The VirB type IV secretion system of *Bartonella henselae* mediates invasion, proinflammatory activation and antiapoptotic protection of endothelial cells. Mol Microbiol 52: 81–92. 15049812 10.1111/j.1365-2958.2003.03964.x

[89] Scheidegger F, Ellner Y, Guye P, Rhomberg TA, Weber H, et al (2009) Distinct activities of *Bartonella henselae* type IV secretion effector proteins modulate capillary-like sprout formation. Cell Microbiol 11: 1088–1101. 19416269 10.1111/j.1462-5822.2009.01313.x

[90] Harms A, Dehio C (2012) Intruders below the radar: molecular pathogenesis of *Bartonella* spp. Clin Microbiol Rev 25: 42–78. 22232371 10.1128/CMR.05009-11 PMC3255967

[91] Kempf VA, Schaller M, Behrendt S, Volkmann B, Aepfelbacher M, et al (2000) Interaction of *Bartonella henselae* with endothelial cells results in rapid bacterial rRNA synthesis and replication. Cell Microbiol 2: 431–441. 11207598 10.1046/j.1462-5822.2000.00072.x

[92] Carroll JA, Coleman SA, Smitherman LS, Minnick MF (2000) Hemin-binding surface protein from *Bartonella quintana* . Infect Immun 68: 6750–6757. 11083791 10.1128/iai.68.12.6750-6757.2000 PMC97776

[93] Battisti JM, Sappington KN, Smitherman LS, Parrow NL, Minnick MF (2006) Environmental signals generate a differential and coordinated expression of the heme receptor gene family of *Bartonella quintana* . Infect Immun 74: 3251–3261. 16714552 10.1128/IAI.00245-06 PMC1479232

[94] Minnick MF, Sappington KN, Smitherman LS, Andersson SG, Karlberg O, et al (2003) Five-member gene family of *Bartonella quintana* . Infect Immun 71: 814–821. 12540561 10.1128/IAI.71.2.814-821.2003 PMC145397

[95] Battisti JM, Smitherman LS, Sappington KN, Parrow NL, Raghavan R, et al (2007) Transcriptional regulation of the heme binding protein gene family of *Bartonella quintana* is accomplished by a novel promoter element and iron response regulator. Infect Immun 75: 4373–4385. 17576755 10.1128/IAI.00497-07 PMC1951173

[96] Dabo SM, Confer AW, Anderson BE, Gupta S (2006) *Bartonella henselae* Pap31, an extracellular matrix adhesin, binds the fibronectin repeat III13 module. Infect Immun 74: 2513–2521. 16622186 10.1128/IAI.74.5.2513-2521.2006 PMC1459717

[97] Parrow NL, Abbott J, Lockwood AR, Battisti JM, Minnick MF (2009) Function, regulation, and transcriptional organization of the hemin utilization locus of *Bartonella quintana* . Infect Immun 77: 307–316. 18981245 10.1128/IAI.01194-08 PMC2612243

[98] Minnick MF (1997) Virulence determinants of *Bartonella bacilliformis*. In: Anderson B, Bendinelli M, Friedman H, editors. Rickettsial Infection and Immunity. New York: Plenum Press. pp. 197–211.

[99] Daly JS, Worthington MG, Brenner DJ, Moss CW, Hollis DG, et al (1993) *Rochalimaea elizabethae* sp. nov. isolated from a patient with endocarditis. J Clin Microbiol 31: 872–881. 7681847 10.1128/jcm.31.4.872-881.1993 PMC263580

[100] Hendrix LR (2000) Contact-dependent hemolytic activity distinct from deforming activity of *Bartonella bacilliformis* . FEMS Microbiol Lett 182: 119–124. 10612742 10.1111/j.1574-6968.2000.tb08884.x

[101] Webster GF, Cockerell CJ, Friedman-Kien AE (1992) The clinical spectrum of bacillary angiomatosis. Br J Dermatol 126: 535–541. 1610703 10.1111/j.1365-2133.1992.tb00096.x

[102] Mohle-Boetani JC, Koehler JE, Berger TG, LeBoit PE, Kemper CA, et al (1996) Bacillary angiomatosis and bacillary peliosis in patients infected with human immunodeficiency virus: clinical characteristics in a case-control study. Clin Infect Dis 22: 794–800. 8722933 10.1093/clinids/22.5.794

[103] Garcia FU, Wojta J, Broadley KN, Davidson JM, Hoover RL (1990) *Bartonella bacilliformis* stimulates endothelial cells in vitro and is angiogenic in vivo. Am J Pathol 136: 1125–1135. 1693472 PMC1877437

[104] Cockerell CJ, LeBoit PE (1990) Bacillary angiomatosis: a newly characterized, pseudoneoplastic, infectious, cutaneous vascular disorder. J Am Acad Dermatol 22: 501–512. 2179301 10.1016/0190-9622(90)70071-o

[105] Noguchi H, Muller HR, Tilden EB, Tyler JR (1929) Etiology of Oroya fever: XVI. Verruga in the dog and the donkey. J Exp Med 50: 455–461. 19869639 10.1084/jem.50.4.455 PMC2131636

[106] Bhutto AM, Nonaka S, Hashiguchi Y, Gomez EA (1994) Histopathological and electron microscopical features of skin lesions in a patient with bartonellosis (verruga peruana). J Dermatol 21: 178–184. 8014271 10.1111/j.1346-8138.1994.tb01717.x

[107] Carmeliet P (2003) Angiogenesis in health and disease. Nat Med 9: 653–660. 12778163 10.1038/nm0603-653

[108] Noguchi H (1926) Etiology of Oroya fever: IV. The effect of inoculation of anthropoid apes with *Bartonella* bacilliformis. J Exp Med 44: 715–728. 19869218 10.1084/jem.44.5.715 PMC2131201

[109] Noguchi H (1926) Etiology of Oroya fever: III. The behavior of *Bartonella bacilliformis* in *Macacus rhesus* . J Exp Med 44: 697–713. 19869217 10.1084/jem.44.5.697 PMC2131202

[110] Infante B, Villar S, Palma S, Merello J, Valencia R, et al (2008) BALB/c Mice resist infection with *Bartonella bacilliformis* . BMC Res Notes 1: 103. 18957122 10.1186/1756-0500-1-103 PMC2590606

[111] Chiaraviglio L, Duong S, Brown DA, Birtles RJ, Kirby J E (2010) An immunocompromised murine model of *Bartonella* infection. Am J Pathol 176: 2753–2763. 20395436 10.2353/ajpath.2010.090862 PMC2877837

[112] Minnick MF, Smitherman LS, Samuels DS (2003) Mitogenic effect of *Bartonella bacilliformis* on human vascular endothelial cells and involvement of GroEL. Infect Immun 71: 6933–6942. 14638782 10.1128/IAI.71.12.6933-6942.2003 PMC308913

[113] McCord AM, Cuevas J, Anderson BE (2007) *Bartonella*-induced endothelial cell proliferation is mediated by release of calcium from intracellular stores. DNA Cell Biol 26: 657–663. 17678436 10.1089/dna.2007.0592

[114] Kirby JE, Nekorchuk DM (2002) *Bartonella*-associated endothelial proliferation depends on inhibition of apoptosis. Proc Natl Acad Sci U S A 99: 4656–4661. 11904386 10.1073/pnas.072292699 PMC123703

[115] Cerimele F, Brown LF, Bravo F, Ihler GM, Kouadio P, et al (2003) Infectious angiogenesis: *Bartonella bacilliformis* infection results in endothelial production of angiopoetin-2 and epidermal production of vascular endothelial growth factor. Am J Pathol 163: 1321–1327. 14507641 10.1016/S0002-9440(10)63491-8 PMC1868281

[116] Pugh CW, Ratcliffe PJ (2003) Regulation of angiogenesis by hypoxia: role of the HIF system. Nat Med 9: 677–684. 12778166 10.1038/nm0603-677

[117] Maxwell PH, Pugh CW, Ratcliffe PJ (2001) Activation of the HIF pathway in cancer. Curr Opin Genet Dev 11: 293–299. 11377966 10.1016/s0959-437x(00)00193-3

[118] Schmid MC, Scheidegger F, Dehio M, Balmelle-Devaux N, Schulein R, et al (2006) A translocated bacterial protein protects vascular endothelial cells from apoptosis. PLoS Pathog 2: e115. 17121462 10.1371/journal.ppat.0020115 PMC1657063

[119] McCord AM, Burgess AW, Whaley MJ, Anderson BE (2005) Interaction of *Bartonella henselae* with endothelial cells promotes monocyte/macrophage chemoattractant protein 1 gene expression and protein production and triggers monocyte migration. Infect Immun 73: 5735–5742. 16113290 10.1128/IAI.73.9.5735-5742.2005 PMC1231114

[120] Ricketts WE (1949) Clinical manifestations of Carrión's disease. Arch Intern Med (Chic) 84: 751–781. 15393033 10.1001/archinte.1949.00230050087005

[121] Knobloch J, Solano L, Alvarez O, Delgado E (1985) Antibodies to *Bartonella bacilliformis* as determined by fluorescence antibody test, indirect haemagglutination and ELISA. Trop Med Parasitol 36: 183–185. 3911360

[122] Howe C (1943) Carrión's disease. Immunologic studies. Arch Intern Med (Chic) 72: 147–167.

[123] Herrer A (1953) Carrión's disease. II. Presence of *Bartonella bacilliformis* in the peripheral blood of patients with the benign tumor form. Am J Trop Med 2: 645–649. 13065632

[124] Guy L, Nystedt B, Toft C, Zaremba-Niedzwiedzka K, Berglund EC, et al (2013) A gene transfer agent and a dynamic repertoire of secretion systems hold the keys to the explosive radiation of the emerging pathogen *Bartonella* . PLoS Genet 9: e1003393. 23555299 10.1371/journal.pgen.1003393 PMC3610622

[125] Seubert A, Falch C, Birtles RJ, Schulein R, Dehio C (2003) Characterization of the cryptic plasmid pBGR1 from *Bartonella grahamii* and construction of a versatile *Escherichia coli*-*Bartonella* spp. shuttle cloning vector. Plasmid 49: 44–52. 12584000 10.1016/s0147-619x(02)00103-8

[126] Saenz HL, Engel P, Stoeckli MC, Lanz C, Raddatz G, et al (2007) Genomic analysis of *Bartonella* identifies type IV secretion systems as host adaptability factors. Nat Genet 39: 1469–1476. 18037886 10.1038/ng.2007.38

[127] Berglund EC, Frank AC, Calteau A, Vinnere Pettersson O, Granberg F, et al (2009) Run-off replication of host-adaptability genes is associated with gene transfer agents in the genome of mouse-infecting *Bartonella grahamii* . PLoS Genet 5: e1000546. 19578403 10.1371/journal.pgen.1000546 PMC2697382

[128] Saisongkorh W, Robert C, La Scola B, Raoult D, Rolain JM (2010) Evidence of transfer by conjugation of type IV secretion system genes between *Bartonella* species and *Rhizobium radiobacter* in amoeba. PLoS ONE 5: e12666. 20856925 10.1371/journal.pone.0012666 PMC2938332

[129] Moran NA (2002) Microbial minimalism: genome reduction in bacterial pathogens. Cell 108: 583–586. 11893328 10.1016/s0092-8674(02)00665-7

[130] Engel P, Salzburger W, Liesch M, Chang CC, Maruyama S, et al (2011) Parallel evolution of a type IV secretion system in radiating lineages of the host-restricted bacterial pathogen *Bartonella* . PLoS Genet 7: e1001296. 21347280 10.1371/journal.pgen.1001296 PMC3037411

[131] Brenner DJ, O'Connor SP, Hollis DG, Weaver RE, Steigerwalt AG (1991) Molecular characterization and proposal of a neotype strain for *Bartonella bacilliformis* . J Clin Microbiol 29: 1299–1302. 1715879 10.1128/jcm.29.7.1299-1302.1991 PMC270104

[132] Birtles RJ, Harrison TG, Fry NK, Saunders NA, Taylor AG (1991) Taxonomic considerations of *Bartonella bacilliformis* based on phylogenetic and phenotypic characteristics. FEMS Microbiol Lett 67: 187–191. 1778432 10.1016/0378-1097(91)90352-b

[133] Birtles RJ, Harrison TG, Taylor AG (1991) The causative agent of bacillary angiomatosis.N Engl J Med. 325: 1447–1448. 10.1056/NEJM199111143252014 1922261

[134] Welch DF, Pickett DA, Slater LN, Steigerwalt AG, Brenner DJ (1992) *Rochalimaea henselae* sp. nov., a cause of septicemia, bacillary angiomatosis, and parenchymal bacillary peliosis. J Clin Microbiol 30: 275–280. 1537892 10.1128/jcm.30.2.275-280.1992 PMC265045

[135] Brenner DJ, O'Connor SP, Winkler HH, Steigerwalt AG (1993) Proposals to unify the genera *Bartonella* and *Rochalimaea*, with descriptions of *Bartonella quintana* comb. nov., *Bartonella vinsonii* comb. nov., *Bartonella henselae* comb. nov., and *Bartonella elizabethae* comb. nov., and to remove the family Bartonellaceae from the order Rickettsiales. Int J Syst Bacteriol 43: 777–786. 8240958 10.1099/00207713-43-4-777

[136] Birtles RJ, Harrison TG, Saunders NA, Molyneux DH (1995) Proposals to unify the genera *Grahamella* and *Bartonella*, with descriptions of *Bartonella talpae* comb. nov., *Bartonella peromysci* comb. nov., and three new species, *Bartonella grahamii* sp. nov., *Bartonella taylorii* sp. nov., and *Bartonella doshiae* sp. nov. Int J Syst Bacteriol 45: 1–8. 7857789 10.1099/00207713-45-1-1

[137] Kosoy M, Hayman DT, Chan KS (2012) *Bartonella* bacteria in nature: where does population variability end and a species start? Infect Genet Evol 12: 894–904. 22449771 10.1016/j.meegid.2012.03.005

[138] La Scola B, Zeaiter Z, Khamis A, Raoult D (2003) Gene-sequence-based criteria for species definition in bacteriology: the *Bartonella* paradigm. Trends Microbiol 11: 318–321. 12875815 10.1016/s0966-842x(03)00143-4

[139] Birtles RJ, Raoult D (1996) Comparison of partial citrate synthase gene (*gltA*) sequences for phylogenetic analysis of *Bartonella* species. Int J Syst Bacteriol 46: 891–897. 8863415 10.1099/00207713-46-4-891

[140] Zeaiter Z, Fournier PE, Ogata H, Raoult D (2002) Phylogenetic classification of *Bartonella* species by comparing *groEL* sequences. Int J Syst Evol Microbiol 52: 165–171. 11837299 10.1099/00207713-52-1-165

[141] Gundi VA, Taylor C, Raoult D, La Scola B (2009) *Bartonella rattaustraliani* sp. nov., *Bartonella queenslandensis* sp. nov. and *Bartonella coopersplainsensis* sp. nov., identified in Australian rats. Int J Syst Evol Microbiol 59: 2956–2961. 19628592 10.1099/ijs.0.002865-0

[142] Inoue K, Kabeya H, Shiratori H, Ueda K, Kosoy MY, et al (2010) *Bartonella japonica* sp. nov. and *Bartonella silvatica* sp. nov., isolated from Apodemus mice. Int J Syst Evol Microbiol 60: 759–763. 19656930 10.1099/ijs.0.011528-0

[143] Alsmark CM, Frank AC, Karlberg EO, Legault BA, Ardell DH, et al (2004) The louse-borne human pathogen *Bartonella quintana* is a genomic derivative of the zoonotic agent *Bartonella henselae* . Proc Natl Acad Sci U S A 101: 9716–9721. 15210978 10.1073/pnas.0305659101 PMC470741

[144] Birtles RJ, Fry NK, Ventosilla P, Cáceres AG, Sánchez E, et al (2002) Identification of *Bartonella bacilliformis* genotypes and their relevance to epidemiological investigations of human bartonellosis. J Clin Microbiol 40: 3606–3612. 12354853 10.1128/JCM.40.10.3606-3612.2002 PMC130851

[145] Hambuch TM, Handley SA, Ellis B, Chamberlin J, Romero S, et al (2004) Population genetic analysis of *Bartonella bacilliformis* isolates from areas of Peru where Carrión's disease is endemic and epidemic. J Clin Microbiol 42: 3675–3680. 15297516 10.1128/JCM.42.8.3675-3680.2004 PMC497641

[146] Chaloner GL, Ventosilla P, Birtles RJ (2011) Multi-locus sequence analysis reveals profound genetic diversity among isolates of the human pathogen *Bartonella bacilliformis* . PLoS Negl Trop Dis 5: e1248. 21811647 10.1371/journal.pntd.0001248 PMC3139668

[147] Tarazona D, Padilla C, Cáceres O, Montenegro JD, Bailón H (2013) Whole Genome Sequencing and Comparative Analysis of *Bartonella bacilliformis* Strain INS, the Causative Agent of Carrión's Disease. Genome Announc 1: e00053–12. 10.1128/genomeA.00053-12 PMC3569277 23409255

[148] Hurtado A, Musso JP, Merino C (1938) La anemia en la enfermedad de Carrión (verruga peruana). An Fac Med Lima 28: 154–168.

[149] Cockerell CJ, Tierno PM, Friedman-Kien AE, Kim KS (1991) Clinical, histologic, microbiologic, and biochemical characterization of the causative agent of bacillary (epithelioid) angiomatosis: a rickettsial illness with features of bartonellosis. J Invest Dermatol 97: 812–817. 1919046 10.1111/1523-1747.ep12487507

[150] Slater LN, Welch DF, Hensel D, Coody DW (1990) A newly recognized fastidious gram-negative pathogen as a cause of fever and bacteremia. N Engl J Med 323: 1587–1593. 2233947 10.1056/NEJM199012063232303

[151] Avidor B, Graidy M, Efrat G, Leibowitz C, Shapira G, et al (2004) *Bartonella koehlerae*, a new cat-associated agent of culture-negative human endocarditis. J Clin Microbiol 42: 3462–3468. 15297484 10.1128/JCM.42.8.3462-3468.2004 PMC497599

[152] Maggi RG, Duncan AW, Breitschwerdt EB (2005) Novel chemically modified liquid medium that will support the growth of seven *Bartonella* species. J Clin Microbiol 43: 2651–2655. 15956379 10.1128/JCM.43.6.2651-2655.2005 PMC1151927

[153] Probert W, Louie JK, Tucker JR, Longoria R, Hogue R, et al (2009) Meningitis due to a “*Bartonella washoensis*”-like human pathogen. J Clin Microbiol 47: 2332–2335. 19439538 10.1128/JCM.00511-09 PMC2708507

[154] Koehler JE, Quinn FD, Berger TG, Leboit PE, Tappero JW (1992) Isolation of *Rochalimaea* species from cutaneous and osseous lesions of bacillary angiomatosis. N Engl J Med 327: 1625–1631. 1435899 10.1056/NEJM199212033272303

[155] Battisti JM, Minnick MF (2008) Laboratory maintenance of *Bartonella quintana*. In: Coico R, Kowalik T, Quarles J, Stevenson B, Taylor R, editors. Current Protocols in Microbiology. New Jersey: John Wiley & Sons. Unit 3C.1.1–3C.1.13. 10.1002/9780471729259.mc03c01s10 18729057

[156] Clarridge JE 3rd, Raich TJ, Pirwani D, Simon B, Tsai L, et al (1995) Strategy to detect and identify *Bartonella* species in routine clinical laboratory yields *Bartonella henselae* from human immunodeficiency virus-positive patient and unique *Bartonella* strain from his cat. J Clin Microbiol 33: 2107–2113. 7559957 10.1128/jcm.33.8.2107-2113.1995 PMC228344

[157] Kreier JP, Gothe R, Ihler GM, Krampitz HE, Mernaugh G, et al. (1992) The hemotrophic bacteria: the families Bartonellaceae and Anaplasmataceae. In: Balows A, Truper HG, Dworkin M, Harder W, Schleifer K-H, editors. The Prokaryotes, A Handbook on the Biology of Bacteria: Ecophysiology, Isolation, Identification, Applications, 2nd edition, New York: Springer-Verlag. pp. 3994–4022.

[158] Knobloch J, Bialek R, Müller G, Asmus P (1988) Common surface epitope of *Bartonella bacilliformis* and *Chlamydia psittaci* . Am J Trop Med Hyg 39: 427–433. 2461660 10.4269/ajtmh.1988.39.427

[159] Minnick MF (1994) Identification of outer membrane proteins of *Bartonella bacilliformis* . Infect Immun 62: 2644–2648. 8188391 10.1128/iai.62.6.2644-2648.1994 PMC186560

[160] Liberto MD, Matera G (2000) Pathogenic mechanisms of *Bartonella quintana* . New Microbiol 23: 449–456. 11061635

[161] Zähringer U, Lindner B, Knirel YA, van den Akker WM, Hiestand R, et al (2004) Structure and biological activity of the short-chain lipopolysaccharide from *Bartonella henselae* ATCC 49882T. J Biol Chem 279: 21046–21054. 14766898 10.1074/jbc.M313370200

[162] Popa C, Abdollahi-Roodsaz S, Joosten LA, Takahashi N, Sprong T, et al (2007) *Bartonella quintana* lipopolysaccharide is a natural antagonist of Toll-like receptor 4. Infect Immun 75: 4831–4837. 17606598 10.1128/IAI.00237-07 PMC2044526

[163] Burgess AWO, Anderson BE (1998) Outer membrane proteins of *Bartonella henselae* and their interaction with human endothelial cells. Microb Pathog 25: 157–164. 9790875 10.1006/mpat.1998.0223

[164] Li DM, Liu QY, Zhao F, Hu Y, Xiao D, et al (2011) Proteomic and bioinformatic analysis of outer membrane proteins of the protobacterium *Bartonella henselae* (Bartonellaceae). Genet Mol Res 10: 1789–1818. 21948745 10.4238/vol10-3gmr1153

[165] Rhomberg TA, Karlberg O, Mini T, Zimny-Arndt U, Wickenberg U, et al (2004) Proteomic analysis of the sarcosine-insoluble outer membrane fraction of the bacterial pathogen *Bartonella henselae* . Proteomics 4: 3021–3033. 15378747 10.1002/pmic.200400933

[166] Boonjakuakul JK, Gerns HL, Chen YT, Hicks LD, Minnick MF, et al (2007) Proteomic and immunoblot analyses of *Bartonella quintana* total membrane proteins identify antigens recognized by sera from infected patients. Infect Immun 75: 2548–2561. 17307937 10.1128/IAI.01974-06 PMC1865797

[167] Eberhardt C, Engelmann S, Kusch H, Albrecht D, Hecker M, et al (2009) Proteomic analysis of the bacterial pathogen *Bartonella henselae* and identification of immunogenic proteins for serodiagnosis. Proteomics 9: 1967–1981. 19333998 10.1002/pmic.200700670

[168] Kordick DL, Swaminathan B, Greene CE, Wilson KH, Whitney AM, et al (1996) *Bartonella vinsonii* subsp. berkhoffii subsp. nov., isolated from dogs; *Bartonella vinsonii* subsp. vinsonii; and emended description of *Bartonella vinsonii* . Int J Syst Bacteriol 46: 704–709. 8782679 10.1099/00207713-46-3-704

[169] Weiss E, Moulder JW (1984) Order I. Rickettsiales *Gieszczkiewicz 1939*. In: Krieg NR, Holt JG, editors. Bergey's Manual of Systematic Bacteriology. Baltimore: Williams & Wilkins. pp. 687–729.

[170] Welch DF, Hensel DM, Pickett DA, San-Joaquin VH, Robinson A, et al (1993) Bacteremia due to *Rochalimaea henselae* in a child: practical identification of isolates in the clinical laboratory. J Clin Microbiol 31: 2381–2386. 8408560 10.1128/jcm.31.9.2381-2386.1993 PMC265765

[171] Breitschwerdt EB, Maggi RG, Lantos PM, Woods CW, Hegarty BC, et al (2010) *Bartonella vinsonii* subsp. berkhoffii and *Bartonella henselae* bacteremia in a father and daughter with neurological disease. Parasit Vectors 3: 29. 20377863 10.1186/1756-3305-3-29 PMC2859367

[172] Breitschwerdt EB, Maggi RG, Sigmon B, Nicholson WL (2007) Isolation of *Bartonella quintana* from a woman and a cat following putative bite transmission. J Clin Microbiol 45: 270–272. 17093037 10.1128/JCM.01451-06 PMC1828989

[173] Relman DA, Loutit JS, Schmidt TM, Falkow S, Tompkins LS (1990) The agent of bacillary angiomatosis: an approach to the identification of uncultured pathogens. N Engl J Med 323: 1573–1580. 2233945 10.1056/nejm199012063232301 PMC13632802

[174] Hadfield TL, Warren R, Kass M, Brun E, Levy C (1993) Endocarditis caused by *Rochalimaea henselae* . Hum Pathol 24: 1140–1141. 8406424 10.1016/0046-8177(93)90196-n

[175] Dauga C, Miras I, Grimont PA (1996) Identification of *Bartonella henselae* and *B. quintana* 16S rDNA sequences by branch-, genus- and species-specific amplification. J Med Microbiol 45: 192–199. 8810946 10.1099/00222615-45-3-192

[176] Anderson B, Sims K, Regnery R, Robinson L, Schmidt MJ, et al (1994) Detection of *Rochalimaea henselae* DNA in specimens from cat scratch disease patients by PCR. J Clin Microbiol 32: 942–948. 8027347 10.1128/jcm.32.4.942-948.1994 PMC263167

[177] Norman AF, Regnery R, Jameson P, Greene C, Krause DC (1995) Differentiation of *Bartonella*-like isolates at the species level by PCR-restriction fragment length polymorphism in the citrate synthase gene. J Clin Microbiol 33: 1797–1803. 7545181 10.1128/jcm.33.7.1797-1803.1995 PMC228273

[178] Kelly TM, Padmalayam I, Baumstark BR (1998) Use of the cell division protein FtsZ as a means of differentiating among *Bartonella* species. Clin Diagn Lab Immunol 5: 766–772. 9801332 10.1128/cdli.5.6.766-772.1998 PMC96199

[179] Bereswill S, Hinkelmann S, Kist M, Sander A (1999) Molecular analysis of riboflavin synthesis genes in *Bartonella henselae* and use of the *ribC* gene for differentiation of *Bartonella* species by PCR. J Clin Microbiol 37: 3159–3166. 10488170 10.1128/jcm.37.10.3159-3166.1999 PMC85516

[180] Colborn JM, Kosoy MY, Motin VL, Telepnev MV, Valbuena G, et al (2010) Improved detection of *Bartonella* DNA in mammalian hosts and arthropod vectors by real-time PCR using the NADH dehydrogenase gamma subunit (nuoG). J Clin Microbiol 48: 4630–4633. 20926707 10.1128/JCM.00470-10 PMC3008469

[181] Rolain JM, Franc M, Davoust B, Raoult D (2003) Molecular detection of *Bartonella quintana*, *B. koehlerae*, *B. henselae*, *B. clarridgeiae*, *Rickettsia felis*, and *Wolbachia pipientis* in cat fleas, France. Emerg Infect Dis 9: 338–342. 12643829 10.3201/eid0903.020278 PMC2958535

[182] Diaz MH, Bai Y, Malania L, Winchell JM, Kosoy MY (2012) Development of a novel genus-specific real-time PCR assay for detection and differentiation of *Bartonella* species and genotypes. J Clin Microbiol 50: 1645–1649. 22378904 10.1128/JCM.06621-11 PMC3347110

[183] Renesto P, Gautheret D, Drancourt M, Raoult D (2000) Determination of the *rpoB* gene sequences of *Bartonella henselae* and *Bartonella quintana* for phylogenic analysis. Res Microbiol 151: 831–836. 11191808 10.1016/s0923-2508(00)01149-9

[184] Minnick MF, Barbian KD (1997) Identification of *Bartonella* using PCR; genus- and species-specific primer sets. J Microbiol Methods 31: 51–57.

[185] Buss SN, Gebhardt LL, Musser KA (2012) Real-time PCR and pyrosequencing for differentiation of medically relevant *Bartonella* species. J Microbiol Methods 91: 252–256. 22960689 10.1016/j.mimet.2012.08.014

[186] Padilla C, Ventura G (2003) Diseño y estandarización de una prueba de PCR para el diagnóstico de la Bartonelosis causada por *Bartonella bacilliformis* . Rev Perú Med Exp Salud Publica 20: 5–8.

[187] Smit PW, Peeling RW, Garcia PJ, Torres LL, Pérez-Lu JE, et al (2013) Dried blood spots for qPCR diagnosis of acute *Bartonella bacilliformis* infection. Am J Trop Med Hyg 89: 988–990. 24043691 10.4269/ajtmh.13-0246 PMC3820349

[188] Westfall HN, Edman DC, Weiss E (1984) Analysis of fatty acids of the genus *Rochalimaea* by electron capture gas chromatography: detection of nonanoic acid. J Clin Microbiol 19: 305–310. 6715507 10.1128/jcm.19.3.305-310.1984 PMC271053

[189] Kordick DL, Wilson KH, Sexton DJ, Hadfield TL, Berkhoff HA, et al (1995) Prolonged *Bartonella* bacteremia in cats associated with cat-scratch disease patients. J Clin Microbiol 33: 3245–3251. 8586710 10.1128/jcm.33.12.3245-3251.1995 PMC228681

[190] Chamberlin J, Laughlin L, Gordon S, Romero S, Solórzano N, et al (2000) Serodiagnosis of *Bartonella bacilliformis* infection by indirect fluorescence antibody assay: test development and application to a population in an area of bartonellosis endemicity. J Clin Microbiol 38: 4269–4271. 11060108 10.1128/jcm.38.11.4269-4271.2000 PMC87581

[191] Padilla C, Gallegos K, Marcelo A, Chenet S, Baldeviano C (2006) Expresión y seroreactividad de la lipoproteína recombinante de 43-kDa de *Bartonella bacilliformis* . Rev Peru Med Exp Salud Publica 23: 182–187.

[192] Mallqui V, Speelmon EC, Verástegui M, Maguiña-Vargas C, Pinell-Salles P, et al (2000) Sonicated diagnostic immunoblot for bartonellosis. Clin Diagn Lab Immunol 7: 1–5. 10618267 10.1128/cdli.7.1.1-5.2000 PMC95812

[193] Maguina C, Garcia PJ, Gotuzzo E, Cordero L, Spach DH (2001) Bartonellosis (Carrión's disease) in the modern era. Clin Infect Dis 33: 772–779. 11512081 10.1086/322614

[194] Maguiña C, Romero I, Soto N, Solórzano N, Tarazona A, et al (2002) Historia natural de la fase eruptiva de la Verruga Peruana y la importancia de la prueba de western blot, reporte preliminar. Fol Dermatol Peru 13: 36–42.

[195] Sobraquès M, Maurin M, Birtles RJ, Raoult D (1999) In vitro susceptibilities of four *Bartonella bacilliformis* strains to 30 antibiotic compounds. Antimicrob Agents Chermother 43: 2090–2092. 10.1128/aac.43.8.2090 PMC89424 10428946

[196] Bartlett JG (2005) 2005–2006 Pocket book of infectious disease therapy. Baltimore: Williams & Wilkins. 349 p.

[197] Rolain JM, Brouqui P, Koehler JE, Maguina C, Dolan MJ, et al (2004) Recommendations for treatment of human infections caused by *Bartonella* species. Antimicrob Agents Chemother 48: 1921–1933. 15155180 10.1128/AAC.48.6.1921-1933.2004 PMC415619

[198] Urteaga O, Payne EH (1955) Treatment of the acute febrile phase of Carrión's disease with chloramphenicol. Am J Trop Med Hyg 4: 507–511. 14376776 10.4269/ajtmh.1955.4.507

[199] Arroyo A (2008) Esquemas de tratamiento para la enfermedad de Carrión no complicada en la ciudad de Caraz, Perú. An Fac Med Lima 69: 7–11.

[200] Biswas S, Raoult D, Rolain JM (2007) Molecular mechanisms of resistance to antibiotics in *Bartonella bacilliformis* . J Antimicrob Chemother 59: 1065–1070. 17449882 10.1093/jac/dkm105

[201] Minnick MF, Wilson ZR, Smitherman LS, Samuels DS (2003) *gyrA* mutations in ciprofloxacin-resistant *Bartonella bacilliformis* strains obtained in vitro. Antimicrob Agents Chemother 47: 383–386. 12499219 10.1128/AAC.47.1.383-386.2003 PMC148966

[202] del Valle LJ, Flores L, Vargas M, García-de-la-Guarda R, Quispe RL, et al (2010) *Bartonella bacilliformis*, endemic pathogen of the Andean region, is intrinsically resistant to quinolones. Int J Infect Dis 14: e506–e510. 19969497 10.1016/j.ijid.2009.07.025

[203] Hodgson CH (1947) The treatment of Carrión's disease with large transfusions. Am J Trop Med Hyg 27: 69–75. 20284814 10.4269/ajtmh.1947.s1-27.1.tms1-270010069

[204] Gutierrez YZ, Luna AS (1998) Verruga peruana tratada con sultamicilina y deflazacort. Dermatol Per 8: 43–46.

[205] Howe C, Hertig M (1943) Prophylactic immunization against Carrión's disease. J Immunol 47: 471–482.

[206] Hertig M, Fairchild GB (1948) The control of *Phlebotomus* in Peru with DDT. Am J Trop Med Hyg 28: 207–230. 18858022 10.4269/ajtmh.1948.s1-28.207

[207] Robert LL, Perich MJ (1995) Phlebotomine sand fly (Diptera: Psychodidae) control using a residual pyrethroid insecticide. J Am Mosq Control Assoc 11: 195–199. 7595445

[208] Alexander B, Usma MC, Cadena H, Quesada BL, Solarte Y, et al (1995) Evaluation of deltamethrin-impregnated bednets and curtains against phlebotomine sandflies in Valle del Cauca, Colombia. Med Vet Entomol 9: 279–283. 7548945 10.1111/j.1365-2915.1995.tb00134.x

[209] Maroli M, Majori G (1991) Permethrin-impregnated curtains against phlebotomine sandflies (Diptera: Psychodidae); laboratory and field studies. Parassitologia 33 Suppl: 399–404. 1841235

[210] Majori G, Maroli M, Sabatinelli G, Fausto AM (1989) Efficacy of permethrin-impregnated curtains against endophilic phlebotomine sandflies in Burkina Faso. Med Vet Entomol 3: 441–444. 2519695 10.1111/j.1365-2915.1989.tb00253.x

[211] Killick-Kendrick R (1999) The biology and control of phlebotomine sand flies. Clin Dermatol 17: 279–289. 10384867 10.1016/s0738-081x(99)00046-2

[212] Schlein Y (1987) Marking of *Phlebotomus papatasi* (Diptera: Psychodidae) by feeding on sprayed, coloured sugar bait: a possible means for behavioural and control studies. Trans R Soc Trop Med Hyg 81: 599. 3445342 10.1016/0035-9203(87)90421-4

[213] Robert LL, Perich MJ, Schlein Y, Jacobson RL, Wirtz RA, et al (1997) Phlebotomine sand fly control using bait-fed adults to carry the larvicide *Bacillus sphaericus* to the larval habitat. J Am Mosq Control Assoc 13: 140–144. 9249650

[214] Mascari TM, Foil LD (2010) Laboratory evaluation of insecticide-treated sugar baits for control of phlebotomine sand flies (Diptera: Psychodidae). J Am Mosq Control Assoc 26: 398–402. 21290935 10.2987/10-6017.1

[215] Yuval B, Schlein Y (1986) Leishmaniasis in the Jordan Valley. III. Nocturnal activity of *Phlebotomus papatasi* (Diptera: Psychodidae) in relation to nutrition and ovarian development. J Med Entomol 23: 411–415. 3735348 10.1093/jmedent/23.4.411

[216] World Health Organization (1968) WHO Interregional traveling seminar on leishmaniasis. Geneva, Switzerland: World Health Organization.

[217] Mascari TM, Clark J, Gordon S, Mitchell MA, Rowton ED, et al (2011) Oral treatment of rodents with insecticides for control of sand flies (Diptera:Psychodidae) and the fluorescent tracer technique (FTT) as a tool to evaluate potential sand fly control methods. J Vector Ecol 36 Supplement 1: S132–S137. 21366765 10.1111/j.1948-7134.2011.00122.x

[218] Mascari TM, Stout RW, Foil LD (2012) Laboratory evaluation of oral treatment of rodents with systemic insecticides for control of bloodfeeding sand flies (Diptera: Psychodidae). Vector Borne Zoonotic Dis 12: 699–704. 22607066 10.1089/vbz.2011.0833

[219] Mascari TM, Stout RW, Foil LD (2013) Oral treatment of rodents with fipronil for feed-through and systemic control of sand flies (Diptera:Psychodidae). J Med Entomol 50: 122–125. 23427660 10.1603/me12157

